# Synthesis of fluoro-functionalized diaryl-λ^3^-iodonium salts and their cytotoxicity against human lymphoma U937 cells

**DOI:** 10.3762/bjoc.14.24

**Published:** 2018-02-07

**Authors:** Prajwalita Das, Etsuko Tokunaga, Hidehiko Akiyama, Hiroki Doi, Norimichi Saito, Norio Shibata

**Affiliations:** 1 Department of Nanopharmaceutical Sciences, Nagoya Institute of Technology, Gokiso, Showa-ku, Nagoya 466-8555, Japan; 2 Faculty of Medical Technology, Fujita Health University, 1-98 Dengakugakubo, Kutsukake-cho, Toyoake, 470-1192, Japan; 3 Pharmaceutical Division, Ube Industries, Ltd. Seavans North Bldg., 1-2-1 Shibaura, Minato-ku, Tokyo 105-8449, Japan; 4 Institute of Advanced Fluorine-Containing Materials, Zhejiang Normal University, 688 Yingbin Avenue, 321004 Jinhua, China

**Keywords:** biological activity, diaryliodonium salt, fluorine, hypervalent iodine, lymphoma, pentafluorosulfanyl

## Abstract

Conscious of the potential bioactivity of fluorine, an investigation was conducted using various fluorine-containing diaryliodonium salts in order to study and compare their biological activity against human lymphoma U937 cells. Most of the compounds tested are well-known reagents for fluoro-functionalized arylation reactions in synthetic organic chemistry, but their biological properties are not fully understood. Herein, after initially investigating 18 fluoro-functionalized reagents, we discovered that the *ortho*-fluoro-functionalized diaryliodonium salt reagents showed remarkable cytotoxicity in vitro. These results led us to synthesize more compounds, previously unknown sterically demanding diaryliodonium salts having a pentafluorosulfanyl (SF_5_) functional group at the *ortho*-position, that is, unsymmetrical *ortho*-SF_5_ phenylaryl-λ^3^-iodonium salts. Newly synthesized mesityl(2-(pentafluoro-λ^6^-sulfanyl)phenyl)iodonium exhibited the greatest potency in vitro against U937 cells. Evaluation of the cytotoxicity of selected phenylaryl-λ^3^-iodonium salts against AGLCL (a normal human B cell line) was also examined.

## Introduction

There has been a surge in the number of reports about fluorine chemistry in recent decades. This is because fluorine is an extremely important element whose presence in a compound can completely change its original physical and chemical characteristics [1–3]. The chemical structures of various pharmaceuticals, agrochemicals and coatings contain fluorine or fluorinated functional groups [4–9]. Therefore, the development of efficient synthetic methodologies for organofluorine compounds has gained much attention [10–15]. Our research group has been actively working in this direction for decades [12–13,16–23]. Our primary goal has been to develop fluorinating and fluoro-functionalized reagents for fluorination [18–19], trifluoromethylation [13,18–19], trifluoromethylthiolation [12,21] and pentafluoroarylation [22–23]. Utilizing these reagents, we have successfully synthesized a wide variety of bioactive organofluorine compounds [24–30] including fluorinated thalidomide (antitumor) [24], fluorinated donepezil (cholinesterase inhibitor) [25], and fluorinated camptothecin (anticancer) [26]. During our research programs focused on the development of novel reagents for fluoro-functionalization [12–13,16–23], as well as the design and synthesis of biologically active fluorine-containing compounds [24–28], we noted that a series of fluoro-functionalization reagents could themselves be highly potential drug candidates. All of the reagents that we developed contain at least one fluorine atom in their structures, which may explain why they have potential biological activity [4–9]. In addition, examination of the successful records of heterocyclic compounds in the pharmaceutical history indicates that some of these reagents have a heterocyclic skeleton which makes them suitable as drug candidates [29–32]. Among these compounds, our group is interested in investigating the biological activity of hypervalent iodine-type reagents [33]. Hypervalent iodine compounds have been receiving a lot of attention lately due to their varied applications in organic synthesis [33–40]. A wide range of bioactive compounds make use of diaryliodonium reagents as a part of their synthesis [41–43]. On the other hand, there are only fragmented reports on the biological activity of diaryliodonium salts [44–49]. Goldstein et al. [45] and Doroshow et al. [46] reported that some diaryliodonium salts show effective antimicrobial and NOX inhibitor activity, respectively. Several aryliodonium salts, aryliodonium ylides, and (diacyloxyiodo)arenes were also examined for their antibacterial activities against ice nucleation active Pseudomonas syringae, and aryliodonium salts, especially those with electron-withdrawing groups, exhibit higher antibacterial activities [49]. Despite the long history of diaryliodonium salts, which exceeds 100 years, as Willgerodt's reagent [50], research on the biological effects of diaryliodonium salts is still undeveloped. Since there are iodine-containing pharmaceuticals such as a series of radiocontrast agents, levothyroxine, idoxuridine and amiodaron [51–54], we started to investigate a biological study of fluorine-containing hypervalent iodine compounds in vitro using U937 (a human histiocytic lymphoma cell line) [55–58]. The U937 cell line is maintained as replicative non-adherent cells having many of the biochemical and morphological characteristics of blood monocytes. This cell line was chosen due to the convenience with which it can be handled and its ease of growth [27]. Initially, 19 compounds [20–22,59–64] were examined for their potential cytotoxicity, and some of them showed potency, in particular *ortho*-fluoro-functionalized diaryliodonium salts. These findings led us to synthesize four more previously unknown diaryliodonium salts having a sterically demanding pentafluorosulfanyl (SF_5_) functional group at the *ortho*-position, that is, unsymmetrical *ortho*-SF_5_ phenylaryl-λ^3^-iodonium salts. Finally, one of the new compounds, namely mesityl(2-(pentafluoro-λ^6^-sulfanyl)phenyl)iodonium salt, exhibited the greatest potency in vitro against U937 cells with an IC_50_ value of 0.49 μM.

## Results and Discussion

**Figure 1:**
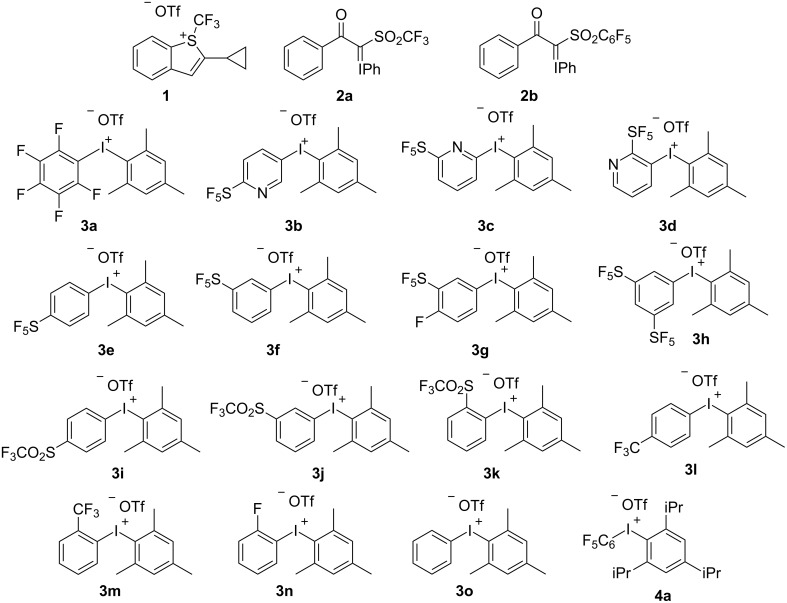
Compounds used for the biological study.

To begin our investigation related to bioactivity, we randomly selected some fluorinating reagents that we had already developed, including Shibata reagents I [20] and II [21] (trifluoromethylation reagent **1** and trifluoromethylthiolation reagent **2a,** respectively), pentafluorophenylating reagent **2b** and several hypervalent iodine reagents, i.e., diaryliodonium salts with a mesitylene ligand (**3a**–**o**) and a triisopropylphenyl ligand **4a** [20–22,59–64] (Figure 1). We used the Muse^TM^ Annexin V and Dead Cell Assay Kit (FITC), which is a common tool to detect the ability of compounds to induce cell death. U937, a human histiocytic lymphoma cell line (DS Pharma Biomedical EC85011440; Osaka, Japan) was used to examine the ability of our synthesized compounds to induce cell death.

**Figure 2:**
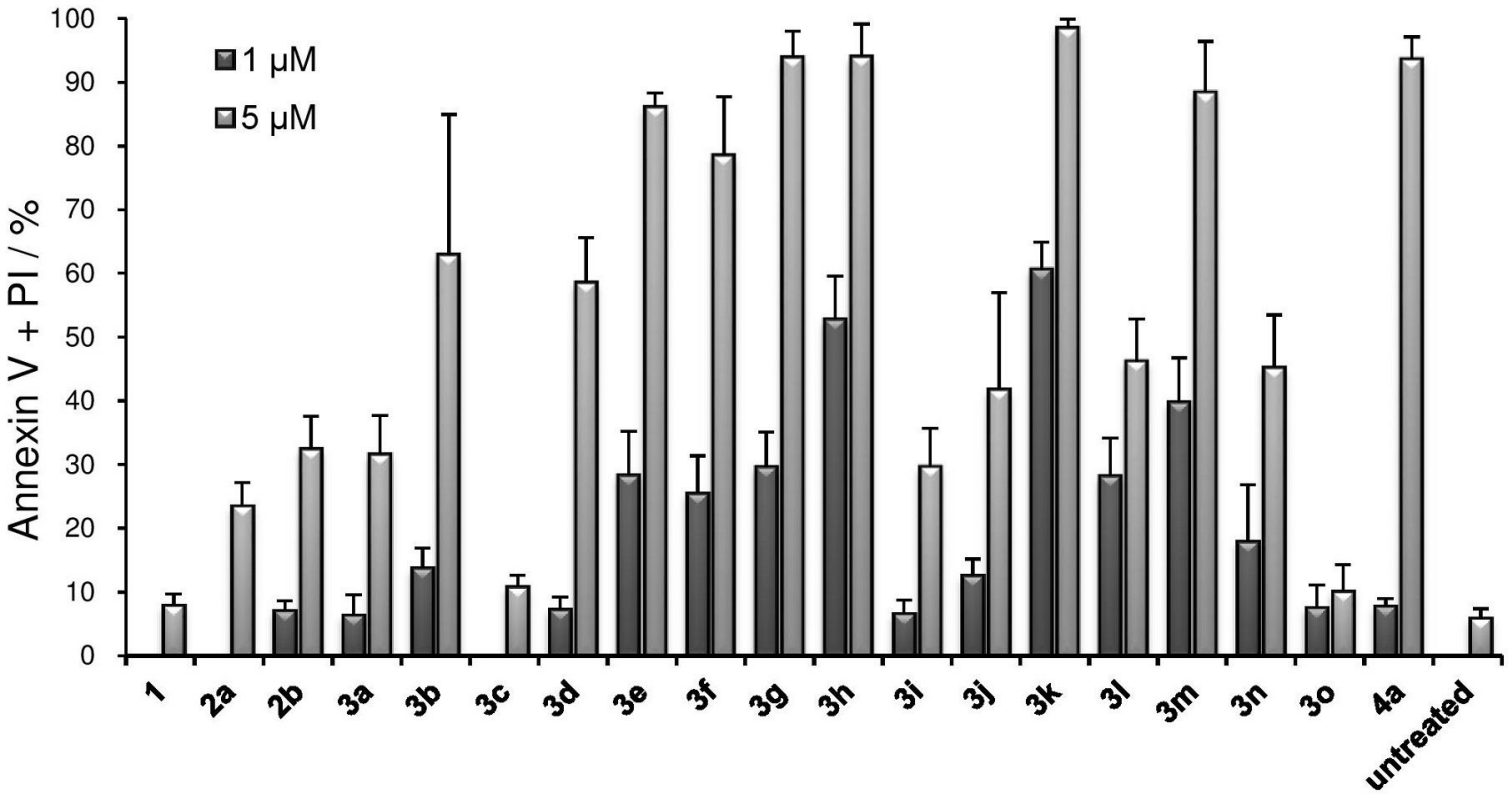
Compounds **1**–**4** induced cell death in U937 cells. Briefly, U937 cells (1 × 10^4^ cells/mL) were incubated with each test compound at 1 μM and 5 μM for 24 h. Cells were stained with annexin V and propidium iodide (PI). The data shown is the mean ± SD (*n* = 3).

The investigation was initially carried out using 20 µM of several of the compounds, all of which showed strong cytotoxicity (Figure S1 in Supporting Information File 1). We therefore opted to examine this potency using lower concentrations (1 µM and 5 µM) of these compounds. The data (treated cells and untreated controls) were plotted together to compare the results (Figure 2). Shibata reagents **1** and **2a** were not very cytotoxic at 5 μM. Perfluorinated phenylthio reagent **2b** and perfluorinated phenyl reagent **3a** showed similar, but unimpressive, results at both concentrations. Pyridinyl reagent **3b**, having a pyridine-SF_5_ moiety, displayed high cytotoxicity (63.0% of annexin V-positive cells) at 5 μM, as did **3d** with 58.6% cytotoxicity. However, another analogue of pyridine-SF_5_ reagent **3c** showed weak (10.8%) cytotoxicity. Next, we investigated the various SF_5_-phenyl aryliodonium salts **3e**–**h**. They all displayed potent cytotoxicities at 5 μM (**3e**, (86.2%); **3f** (78.6%); **3g** (94.0%); **3h** (94.1%)) and **3h** had the greatest result even at 1 μM (52.8%). Thereafter, we analysed SO_2_CF_3_-phenyl aryliodonium salts **3i**–**k** and CF_3_-phenyl aryliodonium salts **3l**,**m**. We observed that *ortho*-substituted phenyl aryliodonium salts **3k** and **3m** seemed to provide great potency at both concentrations (60.6% and 39.8% at 1 μM and 98.6% and 88.4% at 5 μM, respectively). The *ortho*-fluorophenyl aryliodonium salt **3n** was also analysed, and its potency at 5 μM was found to be moderate (45.3%), but low at 1 μM (17.9%). The non-fluorinated diphenyl iodonium salt **3o**, on the other hand, was weakly cytotoxic at both concentrations (1 μM, 7.5%; 5 μM, 10.1%). Analysis of the perfluorinated phenyl reagent **4a** with a triisopropylphenyl ligand displayed strong cytotoxicity (93.7%) at 5 μM, but this value decreased considerably to 7.8% when used at 1 μM.

Following this investigation, we found that the *ortho*-substituted diaryliodonium salts with an *ortho*-SO_2_CF_3_ group **3k** and an *ortho*-CF_3_ group **3m** displayed impressive results. A common feature of *ortho*-substitution on the aromatic group is steric demand, which allowed us to analyse the cell death-inducing potency of phenyl aryliodonium salts with a more sterically demanding fluoro-functional group of SF_5_ at the *ortho*-position. As we did not succeed in synthesizing *ortho*-SF_5_-substituted aryliodonium salts previously [59], we decided to proceed with a further investigation of the synthesis of *ortho*-SF_5_ phenyl aryliodonium salts.

**Scheme 1:**
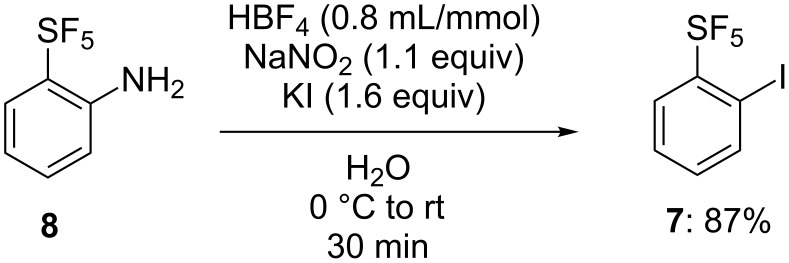
Synthesis of pentafluoro-(2-iodophenyl)-λ^6^-sulfane (**7**).

Four *ortho*-SF_5_-substituted diaryliodonium salts were designed with different arenes as auxiliary groups, namely, electron-rich with sterically demanding mesitylene type **3p** and triisopropylphenyl type **4b**, electron-rich anisole type **5a** and simple phenyl type **6a**, which altered their electronic and steric properties [37]. First, pentafluoro-(2-iodophenyl)-λ^6^-sulfane (**7**) was synthesized from commercially available 2-(pentafluoro-λ^6^-sulfanyl)aniline (**8**), by completing a Sandmeyer reaction (Scheme 1). Fluoroboric acid and sodium nitrite were used to generate the diazonium ion and then KI was used to introduce iodide, providing the desired product **7** in 87% yield.

**Scheme 2:**
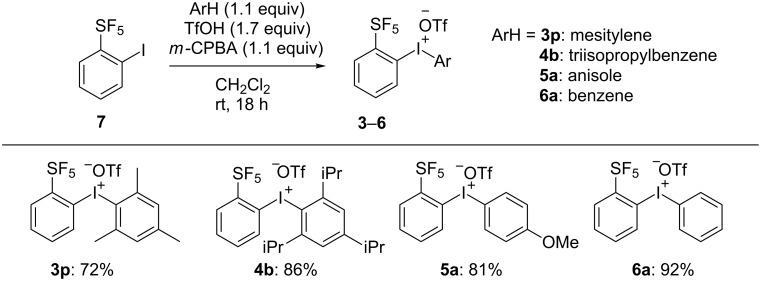
Synthesis of unsymmetrical *ortho*-SF_5_ diaryliodonium salts **3p**, **4b**, **5a** and **6a**.

With **7** in hand, the synthesis of target *ortho*-SF_5_ phenylaryl-λ^3^-iodonium salts **3p**, **4b**, **5a** and **6a** was carried out according to a previously reported method [65–66]. This synthesis was achieved by treating iodide **7** with the respective arene, *m-*CPBA, and trifluoromethanesulfonic acid at room temperature. The desired diaryliodonium salts **3p**, **4b**, **5a** and **6a** were obtained in good yields (72%, 86%, 81% and 92%, respectively) (Scheme 2).

**Figure 3:**
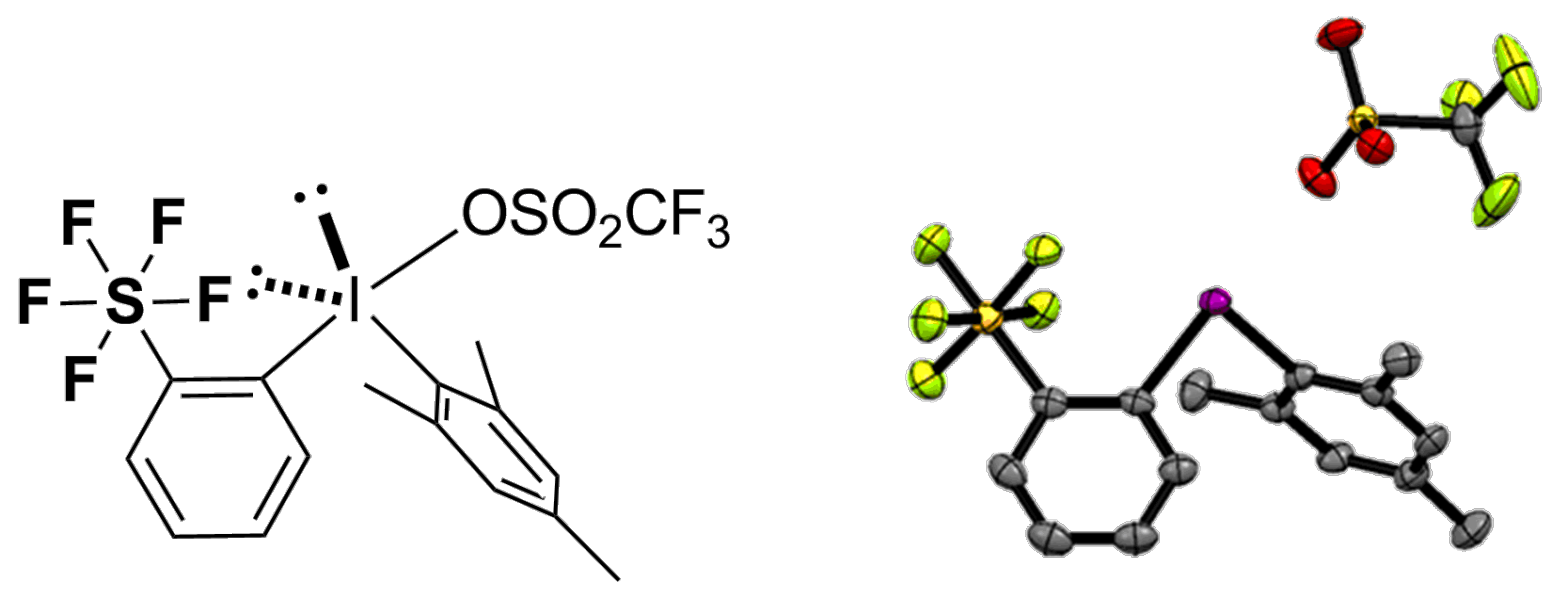
X-ray crystallographic structure of **3p** drawn at 50% probability (CCDC 1573953).

The newly synthesized SF_5_-diaryliodonium salts **3p, 4b, 5a** and **6a** were characterized spectroscopically. The single crystal X-ray structure of **3p** was also analysed. The SF_5_-diaryliodonium salt **3p** has a T-shaped geometry at the iodine centre, consistent with the general structure of diaryliodonium salts [33] (Figure 3).

**Figure 4:**
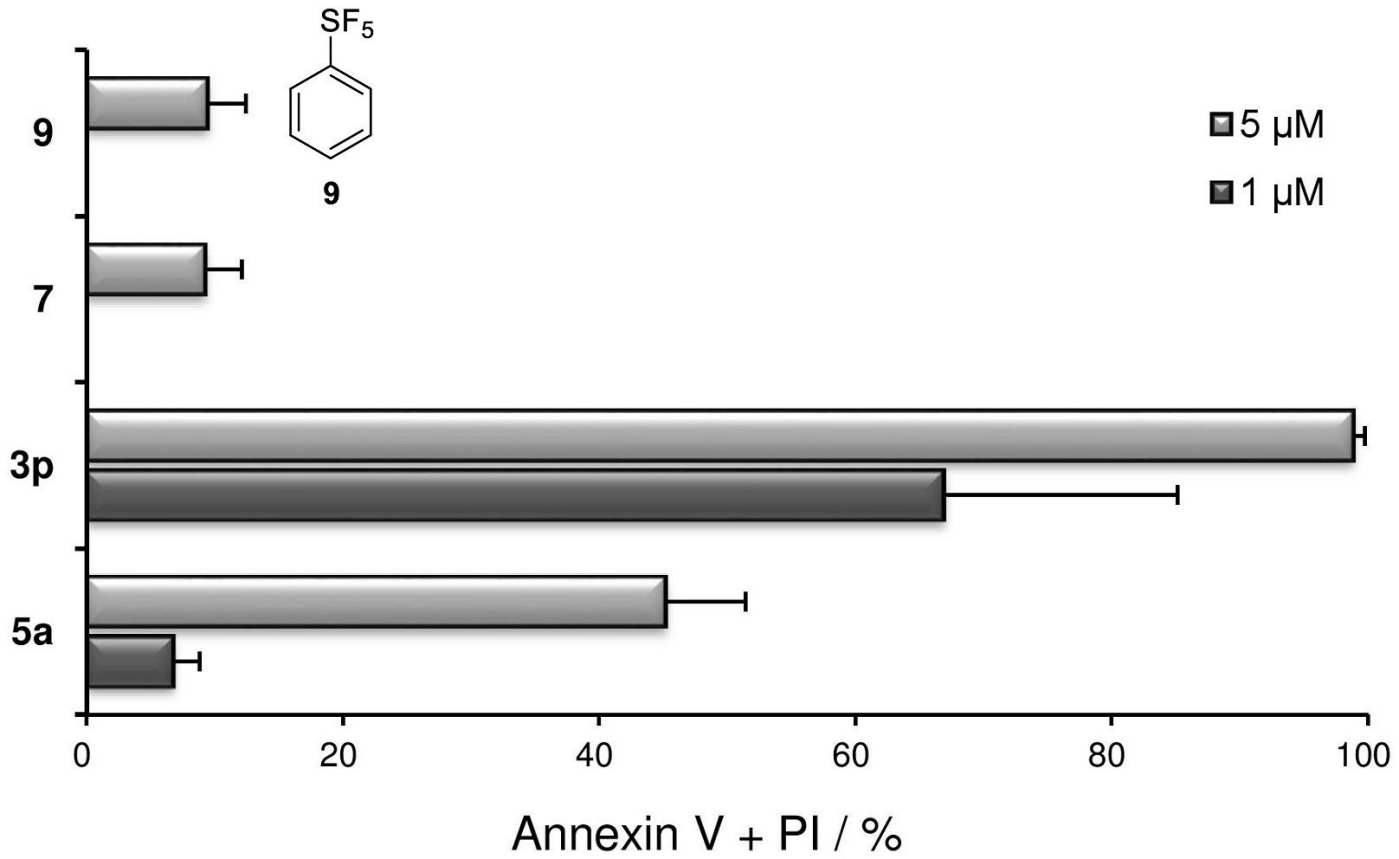
*Ortho*-SF_5_ phenyl iodonium salts **3p** and **5a** and their structural components **7** and **9** induced cell death in U937 cells. Briefly, U937 cells (1 × 10^4^ cells/mL) were incubated with each test compound at 1 μM and 5 μM for 24 h. Cells were stained with annexin V and propidium iodide (PI). The data shown is the mean ± SD (n = 3).

Following the synthesis of the *ortho*-SF_5_ phenyl aryliodonium salts, we selected salts **3p** and **5a** having mesitylene and anisole dummy ligands, respectively, and analysed their potential to induce cell death in U937 cells (Figure 4). Anisole type salt **5a** showed moderate cytotoxicity (45.2%) at 5 μM but the value decreased to 6.8% at 1 μM. Mesitylene type salt **3p**, on the other hand, displayed high potencies (98.9% and 66.9%) at 5 μM and 1 μM, respectively. To ensure that the cytotoxicity of **3p** was not due to its decomposed fragments, we analysed pentafluoro-(2-iodophenyl)-λ^6^-sulfane (**7**) and pentafluoro(phenyl)-λ^6^-sulfane (SF_5_-C_6_H_5_, **9**), which did not exhibit cytotoxicity.

From the above analysis, we selected a series of *ortho*-fluorinated diaryliodonium salts SO_2_CF_3_ type **3k**, CF_3_ type **3m** and SF_5_ type **3p** and examined their IC_50_ values based on an MTT assay (Table 1). While **3k** was more potent than **3m** at both concentrations, i.e., 1 μM and 5 μM (Figure 2), **3m** has a lower IC_50_ value of 0.68 μM than that of **3k** (2.45 μM), as evaluated by the MTT assay. The best potency and IC_50_ value (0.49 μM) was obtained for SF_5_ type **3p**, which is quite impressive when compared to the well-known antitumor drug cytosine arabinoside (ara-C), (0.16 μM) [27].

**Table 1:** Cytotoxicity of diaryliodonium salts **3k**, **3m** and **3p** against a human histiocytic lymphoma cell line (U937).^a^

diaryliodonium salt **3**	IC_50_ [µM]

**3k**	2.45 ± 0.24
**3m**	0.68 ± 0.05
**3p**	0.49 ± 0.05

*^a^*IC_50_ values were determined using an MTT assay; data represents the mean standard deviation of three independent experiments.

**Figure 5:**
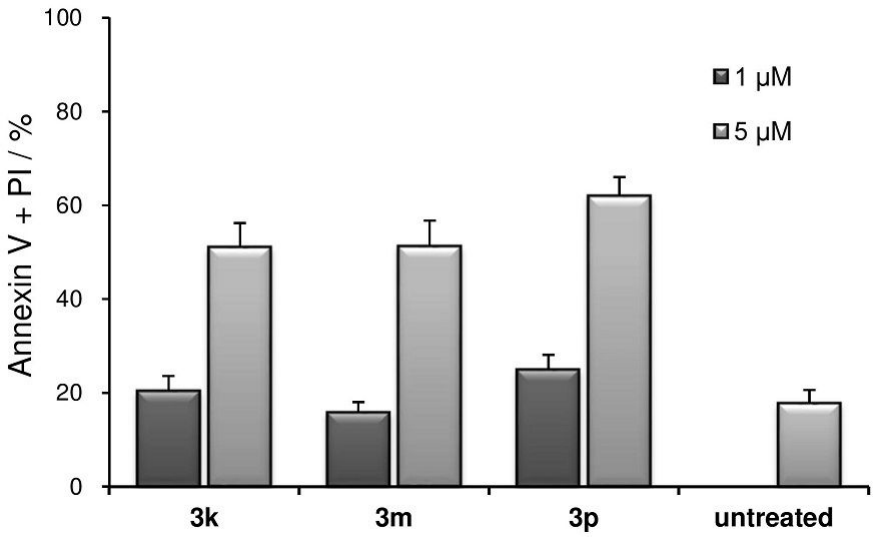
**3k**, **3m** and **3p** induced cell death in AGLCL, a human normal B cell line. Briefly, AGLCL cells (1 × 10^4^ cells/mL) were incubated with each test compound at 1 μM and 5 μM for 24 h. Cells were stained with annexin V and propidium iodide (PI). The data shown is the mean ± SD (*n* = 3).

Since, **3k**, **3m** and **3p** exhibited strong cytotoxicity against U937 cells, we finally evaluated their cytotoxicity against normal cells in vitro. AGLCL, a human normal B cell line (DS Pharma Biomedical EC89120566; Osaka, Japan) was chosen for the experiments and investigations were performed at 20 μM (Figure S2 in Supporting Information File 1) and 5 μM and 1 μM concentrations (Figure 5) of the compounds. Although **3k**, **3m** and **3p** exhibited cytotoxicity even against AGLCL cells, a remarkable difference was observed. That is, moderate cytotoxicity at 5 μM (51.1%, 51.2% and 62.0%, respectively) and low cytotoxicity at 1 μM concentration (20.4%, 15.8% and 24.9%, respectively) against AGLCL cells were observed. It is noteworthy that the cytotoxicity displayed by **3k**, **3m** and **3p** against U937 cells is much higher than those against AGLCL cells at both concentrations. These results strongly suggested that antitumor drug candidates could be designed by further structural modification of these compounds **3**. Moreover, with **3p** exhibiting the greatest potency against U937 cells with comparably lower toxicity against AGLCL cells, further biological studies using **3p** including in vivo evaluation should be conducted. A mechanistic study that examines the structure–cytotoxicity relationships of a series of diaryliodonium salts **3** will also be conducted.

## Conclusion

In conclusion, we have analysed a series of fluorinating reagents and diaryliodonium salts for their applicability in inducing cell death based on U937 (a human histiocytic lymphoma cell line). We have also successfully synthesized novel *ortho*-SF_5_ phenylaryl-λ^3^-iodonium salts. As expected, several fluorinated diaryliodonium salts exhibited cytotoxicity. Among the series, the newly synthesized *ortho*-SF_5_ salt **3p** displayed the greatest potency. The *ortho*-fluorinated diaryliodonium salts **3k**, **3m** and **3p** were also examined for comparison studies with AGLCL (a normal human B cell line). Although the values were rather low, selectivity was indeed observed against U937 cells. Also, even though the results are in a preliminary stage of biological evaluation, this is the first report to highlight the cytotoxicity of diaryliodonium salts against U937 cells. Since diaryliodonium salts are fundamentally oxidizing agents, there might be a stronger correlation between cytotoxicity and the oxidation potential of these salts. We will continue the biological investigation of **3** in this direction. From the view point of organic synthesis, the newly synthesized *ortho*-SF_5_-substituted unsymmetrical iodonium salts **3p**, **4b**, **5a** and **6a** have potential use as electrophilic SF_5_-phenylation reagents for a range of nucleophiles such as alcohols, amines, thiols, and active methylene nucleophiles [59–61]. The application of these *ortho*-SF_5_-substituted diaryliodonium salts in organic synthesis, as well as their detailed bioactive behaviour, will be reported in due course.

## Experimental

### Biological assay

**Quantification of cytotoxicity by annexin V and propidium iodide (PI)**: Cytotoxicity was detected with the Muse^TM^ Annexin V and Dead Cell Assay Kit (Merck Millipore Corp., Darmstadt, Germany) and Muse Cell Analyzer (Merck Millipore Corp.) according to the manufacturer’s protocols. Cells incubated in the presence or absence of the fluorinated compounds for 24 h were collected by centrifugation (2,000 rpm at 4 °C for 5 min). Cells were suspended in 100 μL of RPMI 1640 medium (Sigma-Aldrich, Steinheim, Germany), and incubated with 100 μL of annexin V reagent (in the kit) at room temperature for 20 min. These cells were measured by the Muse Cell Analyzer.

**Statistical analysis:** Data were analyzed using Excel software. Results are expressed as the mean ± SD of three independent replicates.

**MTT assay:** The 3-(4,5-dimethylthiazol-2-yl)-2,5-diphenyltetrazolium bromide (MTT) assay was performed to evaluate cell viability by diaryliodonium salt compounds using the MTT cell proliferation assay kit (Cayman Chemical Company, Ann Arbor, USA). U937 cells were incubated in solutions containing the diaryliodonium salts (**3k, 3m** or **3p**). After this treatment, the U937 cells were seeded in culture medium (100 μL) in a 96-well plate (Becton and Dickinson, at a density of 2 × 10^5^ cells/well) and incubated at 37 °C for 24 h. MTT reagent (10 μL) was added to each well. After mixing gently, the cells were incubated for 3 h at 37 °C in a CO_2_ incubator. The culture medium was aspirated and the crystal-dissolving solution (100 μL) was added to each well and mixed. Finally, the optical density was measured (550 nm) using a microplate reader (BIO-RAD, Benchmark, Hercules, USA).

### General information

All reactions were performed in oven-dried glassware under positive pressure of nitrogen or argon unless mentioned otherwise. Solvents were transferred via a syringe and were introduced into reaction vessels though a rubber septum. All reactions were monitored by thin-layer chromatography (TLC) carried out on 0.25 mm Merck silica gel (60-F254). The TLC plates were visualized with UV light (254 nm). Column chromatography was carried out on columns packed with silica gel (60N spherical neutral size 63–210 μm). The ^1^H NMR (300 MHz), ^19^F NMR (282 MHz), and ^13^C NMR (125 MHz) spectra for solution in CDCl_3_ or (CD_3_)_2_CO were recorded on Varian Mercury 300 and Bruker Avance 500 spectrometers. Chemical shifts (δ) are expressed in ppm downfield from TMS (δ = 0.00) or C_6_F_6_ [δ = −162.2 (CDCl_3_) or −163.5 ((CD_3_)_2_CO)] as an internal standard. Mass spectra were recorded on a Shimadzu GCMS-QP5050A (EIMS) and Shimazu LCMS-2020 (ESIMS) spectrometer. The solvent CH_2_Cl_2_ was dried and distilled before use.

**Preparation of pentafluoro(2-iodophenyl)-λ^6^-sulfane (7):** The preparation of **7** was based on a modified procedure in the literature [67]. To 4 mL of HBF_4_ in a round bottomed flask, 1.1 g of 2-(pentafluoro-λ^6^-sulfanyl)aniline was added and heated until a clear solution formed. The solution was cooled to 0 °C and a cold solution of NaNO_2_ (380 mg in 2.5 mL distilled water) was added dropwise. The reaction was allowed to stir at 0 °C for 15 min after which it was added dropwise to a cold stirred solution of KI (1.33 g in 10 mL distilled water) at 0 °C. The reaction was allowed to warm to room temperature then stirred for 20 min. The reaction mixture was extracted with diethyl ether (3 × 20 mL). The combined organic extract was washed with NaHCO_3_ solution and Na_2_S_2_O_3_ solution and dried over Na_2_SO_4_. The solvent was concentrated under reduced pressure to give a crude product which was purified using silica gel column chromatography (9:1, hexane/ethyl acetate) to give 1.4 g of **7** as a yellow oil in 87% yield. HRMS (EI–TOF) *m*/*z* [M]^+^: calcd for C_6_H_4_F_5_SI, 329.8999; found, 329.9010; ^1^H NMR (CDCl_3_, 300 MHz) δ 7.11 (t, *J* = 9 Hz, 1H), 7.41–7.47 (m, 1H), 7.80 (dd, *J* = 9 Hz, 3 Hz, 1H), 8.14 (d, *J* = 9 Hz, 1H); ^19^F NMR (CDCl_3_, 282 MHz) δ 63.55 (d, *J* = 155.1 Hz, 4F), 83.56 (q, *J* = 155.1 Hz, 1F); ^13^C{^1^H}NMR (CDCl_3_, 126 MHz) δ 88.4, 128.1, 130.3 (t, *J* = 3.75 Hz), 132.4, 144.1, 158.57 (q, *J* = 15 Hz).

#### General procedure A: preparation of diaryliodonium salts I

These salts were prepared according to a modified procedure in the literature [64–65]. *m-*CPBA (assume 70 wt %, 1.1 equiv) was dried in vacuo at room temperature for 1 h before the addition of **7** (1.0 equiv) and CH_2_Cl_2_ (6.0 mL/mmol ArI) in a round-bottomed flask. The solution was cooled to 0 °C followed by the dropwise addition of TfOH (1.7 equiv). The resulting mixture was stirred at room temperature for 2 h. It was then cooled to 0 °C and the arene (1.1 equiv) was added dropwise. The mixture was warmed to room temperature and stirred for 18 h. The solvent was then removed under reduced pressure. The resulting crude product was precipitated by the addition of Et_2_O. The precipitate was filtered and dried in vacuo to give **3**–**6** as an off-white to white solid.

**Mesityl(2-(pentafluoro-λ^6^-sulfanyl)phenyl)iodonium trifluoromethanesulfonate (3p)***:* Following general procedure A, **7** (330 mg, 1 mmol), *m-*CPBA (271 mg, 1.1 mmol), TfOH (0.2 mL, 1.7 mmol) and mesitylene (0.15 mL, 1.1 mmol) in CH_2_Cl_2_ (6 mL) were used from 0 °C to room temperature for 18 h to give **3p** as a white solid (430 mg) in 72% yield. mp: 163.7–164.7 °C; HRMS (ESI–TOF) *m*/*z* [M − OTf]^+^: calcd for C_15_H_15_F_5_SI, 448.9859; found, 448.9865; ^1^H NMR ((CD_3_)_2_CO), 300 MHz) δ 2.44 (s, 3H), 2.69 (s, 6H), 7.42 (s, 2H), 7.64 (d, *J* = 6 Hz, 1H), 7.73 (t, *J* = 9 Hz, 1H), 7.96 (t, *J* = 9 Hz, 1H), 8.36 (dd, *J* = 9 Hz, 1.5 Hz, 1H); ^19^F NMR ((CD_3_)_2_CO), 282 MHz) δ = –79.88 (s, 3F), 64.09 (d, *J* = 149.5 Hz, 4F), 81.34 (q, *J* = 149.5 Hz, 1F); ^13^C{^1^H}NMR ((CD_3_)_2_CO), 126 MHz) δ 21.2, 27.1, 106.6, 121.9 (q, *J* = 318.8 Hz), 123.0, 131.8, 132.5 (t, *J* = 5 Hz), 133.7, 135.4, 136.9, 144.5, 147.0, 154.1–154.7 (m).

**(2-(Pentafluoro-λ^6^-sulfanyl)phenyl)(2,4,6-triisopropylphenyl)iodonium trifluoromethanesulfonate (4b)**: Following general procedure A, **7** (330 mg, 1 mmol), *m-*CPBA (271 mg, 1.1 mmol), TfOH (0.2 mL, 1.7 mmol) and triisopropylbenzene (0.26 mL, 1.1 mmol) in CH_2_Cl_2_ (6 mL) were used from 0 °C to room temperature for 18 h to give **4b** as a white solid (604 mg) in 86% yield. mp: 106.6–107.9 °C; HRMS (ESI–TOF) *m*/*z* [M − OTf]^+^: calcd for C_21_H_27_F_5_SI, 533.0798; found, 533.0798; ^1^H NMR ((CD_3_)_2_CO, 300 MHz) δ 1.31 (t, *J* = 9 Hz, 18H), 3.14 (q, *J* = 9 Hz, 1H), 3.30 (q, *J* = 6 Hz, 2H), 7.45 (d, *J* = 9 Hz, 1H), 7.58 (s, 2H), 7.77 (t, *J* = 6 Hz, 1H), 7.96 (t, *J* = 9 Hz, 1H), 8.37 (dd, *J* = 9 Hz, 1.5 Hz, 1H); ^19^F NMR ((CD_3_)_2_CO, 282 MHz) δ −79.91 (s, 3F), 64.02 (d, *J* = 149.5 Hz, 4F), 81.31 (q, *J* = 149.5 Hz, 1F); ^13^C{^1^H}NMR ((CD_3_)_2_CO, 126 MHz) δ 23.8, 24.2, 34.9, 40.8, 107.7, 121.9 (q, *J* = 320 Hz), 123.5, 127.1, 132.7 (t, *J* = 5 Hz), 133.7, 134.5, 136.9, 153.8, 153.9–154.2 (m), 158.1.

**(4-Methoxyphenyl)(2-(pentafluoro-λ^6^-sulfanyl)phenyl)iodonium trifluoromethanesulfonate (5a)**: Following general procedure A, **7** (416 mg, 1.26 mmol), *m-*CPBA (340 mg, 1.38 mmol), TfOH (0.24 mL, 2.14 mmol) and anisole (0.15 mL, 1.38 mmol) in CH_2_Cl_2_ (6 mL) were used from 0 °C to room temperature for 18 h to give **5a** as a white solid (600 mg) in 81% yield. mp: 108.7–110.4 °C; HRMS (ESI–TOF) *m*/*z* [M − OTf]^+^: calcd for C_13_H_11_OF_5_SI, 436.9495; found, 436.9499; ^1^H NMR ((CD_3_)_2_CO, 300 MHz) δ 3.90 (s, 3H), 7.16 (d, *J* = 9 Hz, 2H), 7.86 (t, *J* = 9 Hz, 1H), 8.03 (t, *J* = 9 Hz, 1H), 8.29 (d, *J* = 9 Hz, 3H), 8.92 (d, *J* = 6 Hz, 1H); ^19^F NMR ((CD_3_)_2_CO, 282 MHz) δ −79.75 (s, 3F), 65.79 (d, *J* = 149.5 Hz, 4F), 81.73 (q, *J* = 152.3 Hz, 1F); ^13^C{^1^H}NMR ((CD_3_)_2_CO, 126 MHz) δ 56.4, 104.8, 109.8, 118.9, 131.7, 134.6, 136.9, 138.8, 142.2, 154.1–154.4 (m), 164.5.

**(2-(Pentafluoro-λ^6^-sulfanyl)phenyl)(phenyl)iodonium trifluoromethanesulfonate (6a)**: Following general procedure A, **7** (330 mg, 1 mmol), *m-*CPBA (271 mg, 1.1 mmol), TfOH (0.2 mL, 1.7 mmol) and benzene (0.1 mL, 1.1 mmol) in CH_2_Cl_2_ (6 mL) were used from 0 °C to room temperature for 18 h to give **6a** as a white solid (512 mg) in 92% yield. mp: 109.8–111.3 °C; HRMS (ESI–TOF) *m*/*z* [M–OTf]^+^: calcd for C_12_H_9_F_5_SI, 406.9390; found, 406.9385; ^1^H NMR ((CD_3_)_2_CO, 300 MHz) δ 7.61–7.68 (m, 2H), 7.76–7.82 (m, 1H), 7.89 (t, *J* = 9 Hz, 1H), 8.06 (t, *J* = 9 Hz, 1H), 8.31 (dd, *J* = 9 Hz, 3 Hz, 1H), 8.37 (d, *J* = 9 Hz, 2H), 9.02 (d, *J* = 6 Hz, 1H); ^19^F NMR ((CD_3_)_2_CO, 282 MHz) δ −79.81 (s, 3F), 65.83 (d, *J* = 149.5 Hz, 4F), 81.61 (q, *J* = 152.3 Hz, 1F); ^13^C{^1^H}NMR ((CD_3_)_2_CO, 126 MHz) δ 109.1, 116.9, 121.9 (q, *J* = 320 Hz), 131.8, 133.2, 134.1, 134.8, 136.3, 136.9, 142.9, 154.3–154.9 (m).

## Supporting Information

File 1 Cytotoxicity data, copies of ^1^H, ^19^F and ^13^C NMR spectra of **7**, **3p**, **4b**, **5a** and **6a** and the ORTEP diagram of **3p**.
