# Supplementary material for: Synthesis of fluoro-functionalized diaryl-λ3-iodonium salts and their cytotoxicity against human lymphoma U937 cells
Source: Beilstein J Org Chem. 2018 Feb 7;14:364–72. doi: 10.3762/bjoc.14.24 (PMC5815272; doi:10.3762/bjoc.14.24)
Supplement: File 1 — Cytotoxicity data, copies of 1H, 19F and 13C NMR spectra of 7, 3p, 4b, 5a and 6a and the ORTEP diagram of 3p. [file Beilstein_J_Org_Chem-14-364-s001.pdf]

Supporting Information

for

Synthesis of fluoro-functionalized diaryl-λ^3-iodonium salts and their cytotoxicity against human lymphoma U937 cells

Prajwalita Das[‡1], Etsuko Tokunaga[‡1], Hidehiko Akiyama[2], Hiroki Doi[2], Norimichi Saito[3], and Norio Shibata*[1,4]

Address: [1]Department of Nanopharmaceutical Sciences, Nagoya Institute of Technology, Gokiso, Showa-ku, Nagoya 466-8555, Japan, [2]Faculty of Medical Technology, Fujita Health University, 1-98 Dengakugakubo, Kutsukake-cho, Toyoake, 470-1192, Japan, [3]Pharmaceutical Division, Ube Industries, Ltd. Seavans North Bldg., 1-2-1 Shibaura, Minato-ku, Tokyo 105-8449, Japan and [4]Institute of Advanced Fluorine-Containing Materials, Zhejiang Normal University, 688 Yingbin Avenue, 321004 Jinhua, China

Email: Norio Shibata* - nozshiba@nitech.ac.jp

*Corresponding author

‡Equal contributors

Cytotoxicity data, copies of ^{1}H, ^{19}F and ^{13}C NMR spectra of 7, 3p, 4b, 5a and 6a and the ORTEP diagram of 3p

Table of Contents

Cytotoxicity against U937 cell line at 20 µM concentration S1

Cytotoxicity against AGLCL cell line at 20 µM concentration S2

Ortep diagram of **3p** S3

NMR Spectra S4

Evaluation of the cytotoxicity of diaryliodonium salts against Human leukemic monocyte lymphoma U937 at 20 µM concentration:

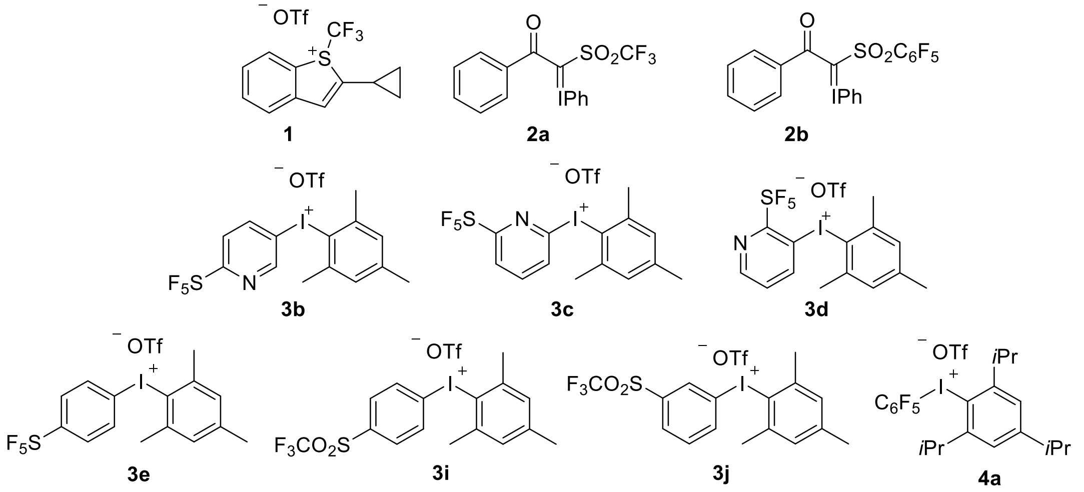

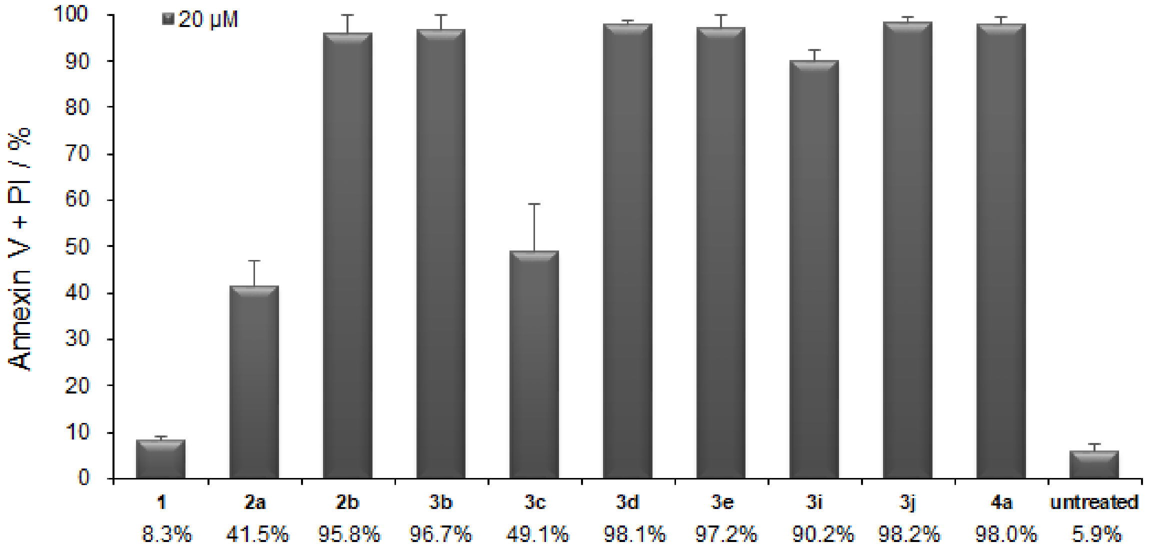

Figure S1: Compounds **1–4** induced cell death in U937 cells. Briefly, U937 cells (1x10^4 cells/mL) were incubated with each test compound at 20 µM for 24 h. Cells were stained with annexin V and propidium iodide (PI). The data is shown as mean ± SD (n = 3).

Evaluation of cytotoxicity of *ortho*-fluorinated diaryliodonium salts **3k**, **3m** and **3p** towards human normal B AGLCL cell line at 20 µM concentration:

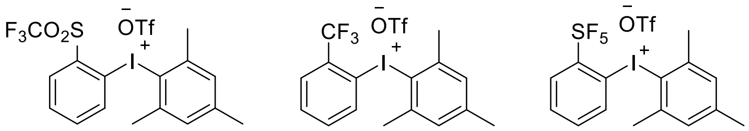

3k **3m** **3p**

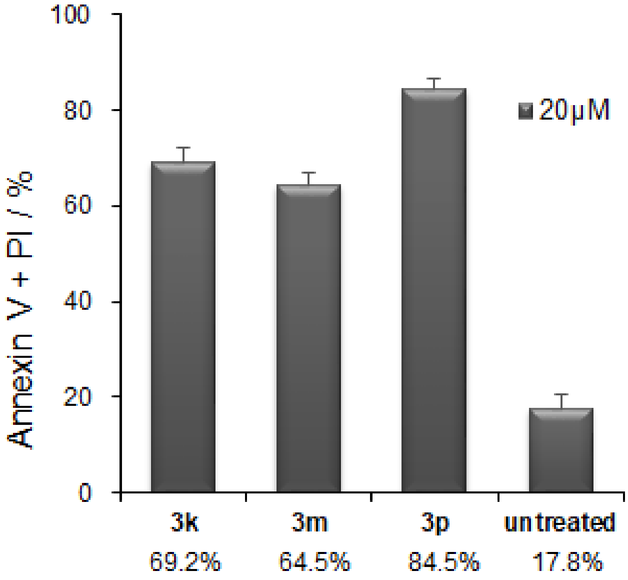

Figure S2: **3k**, **3m** and **3p** induced cell death in AGLCL, a human normal B cell line ($1x10^4$ cells/mL) were incubated with each test compound at 20 µM for 24 h. Cells were stained with annexin V and propidium iodide (PI). The data is shown as mean ± SD (n = 3).

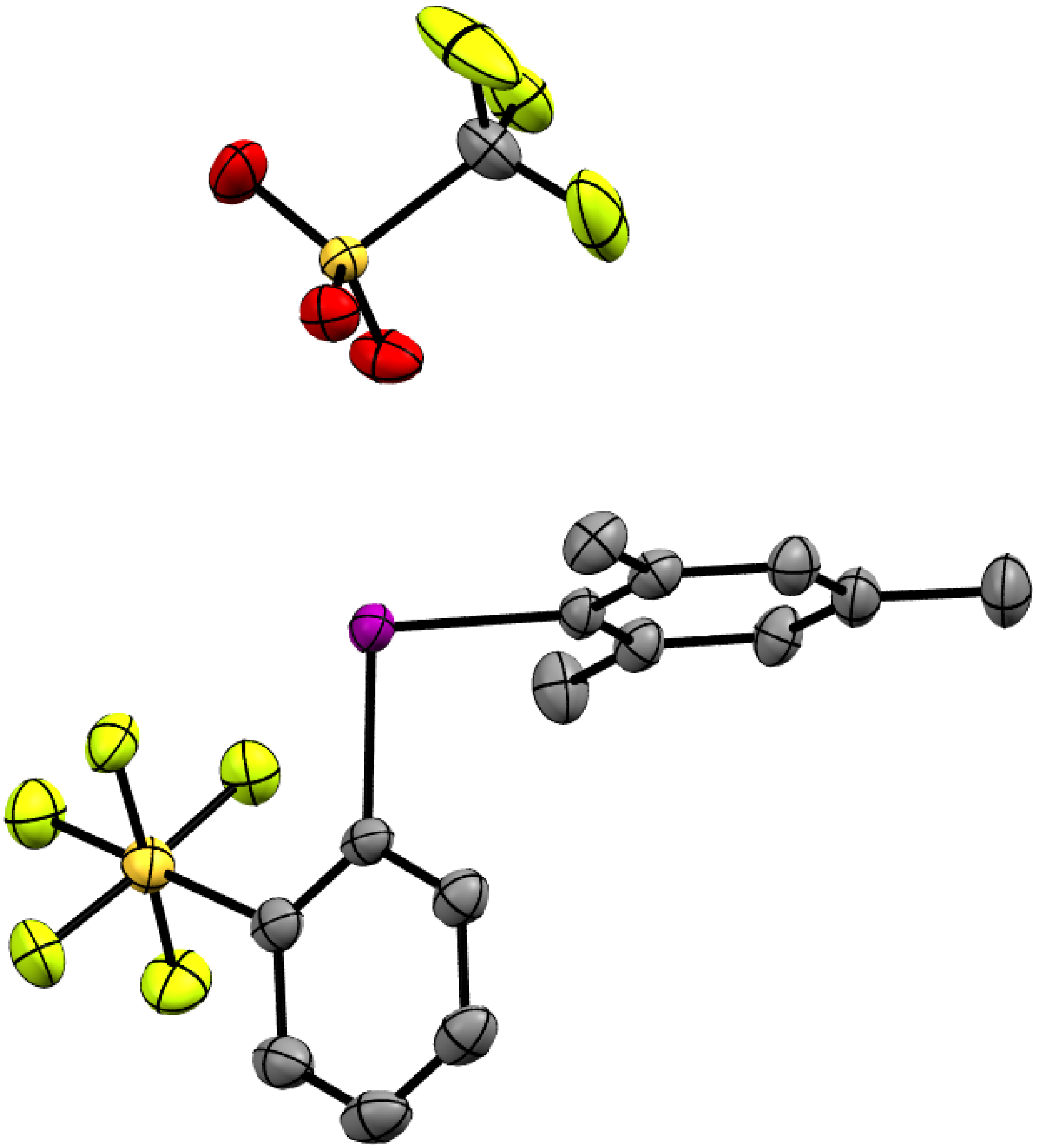

Figure 1: Ortep diagram of **3p** drawn at 50% probability. The hydrogen atoms have been omitted for clarity.

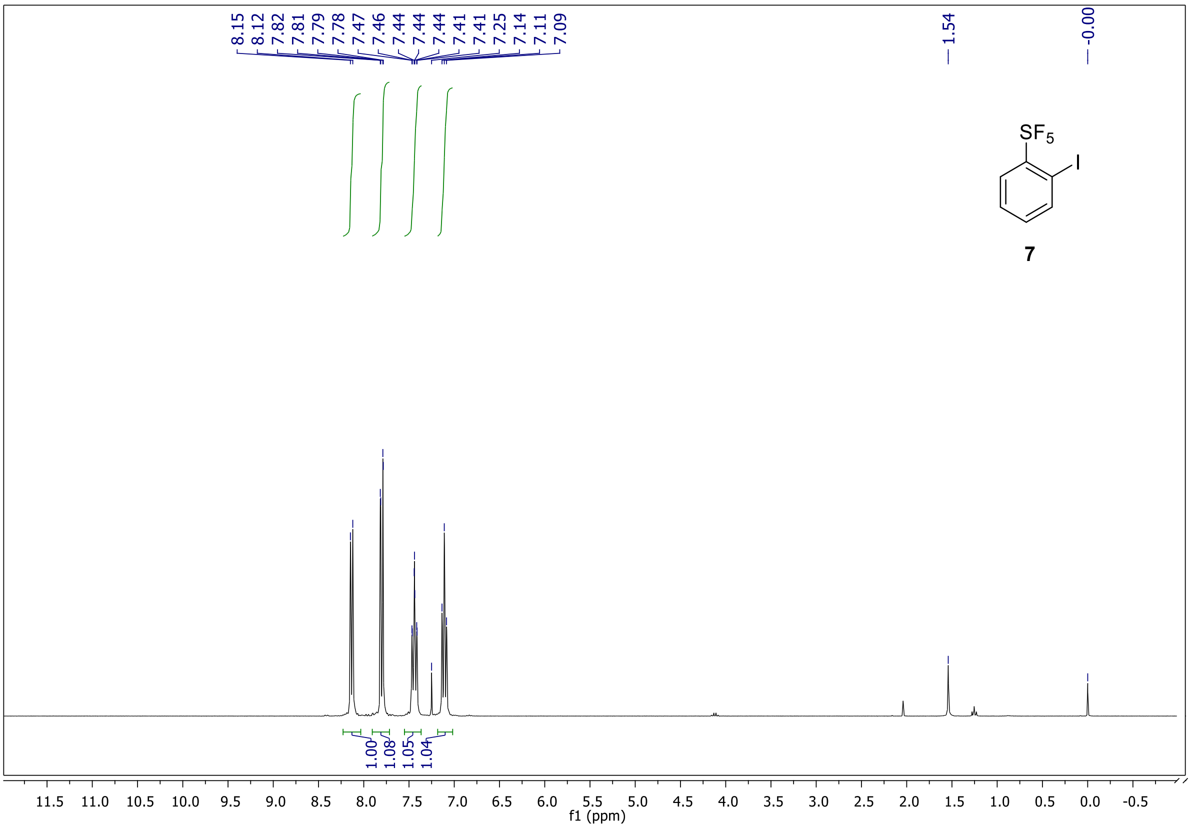
8.15
8.12
7.82
7.81
7.79
7.78
7.47
7.46
7.44
7.44
7.44
7.41
7.41
7.25
7.14
7.11
7.09
1.54
-0.00
SF5
I
7
1.00
1.08
1.05
1.04
f1 (ppm)

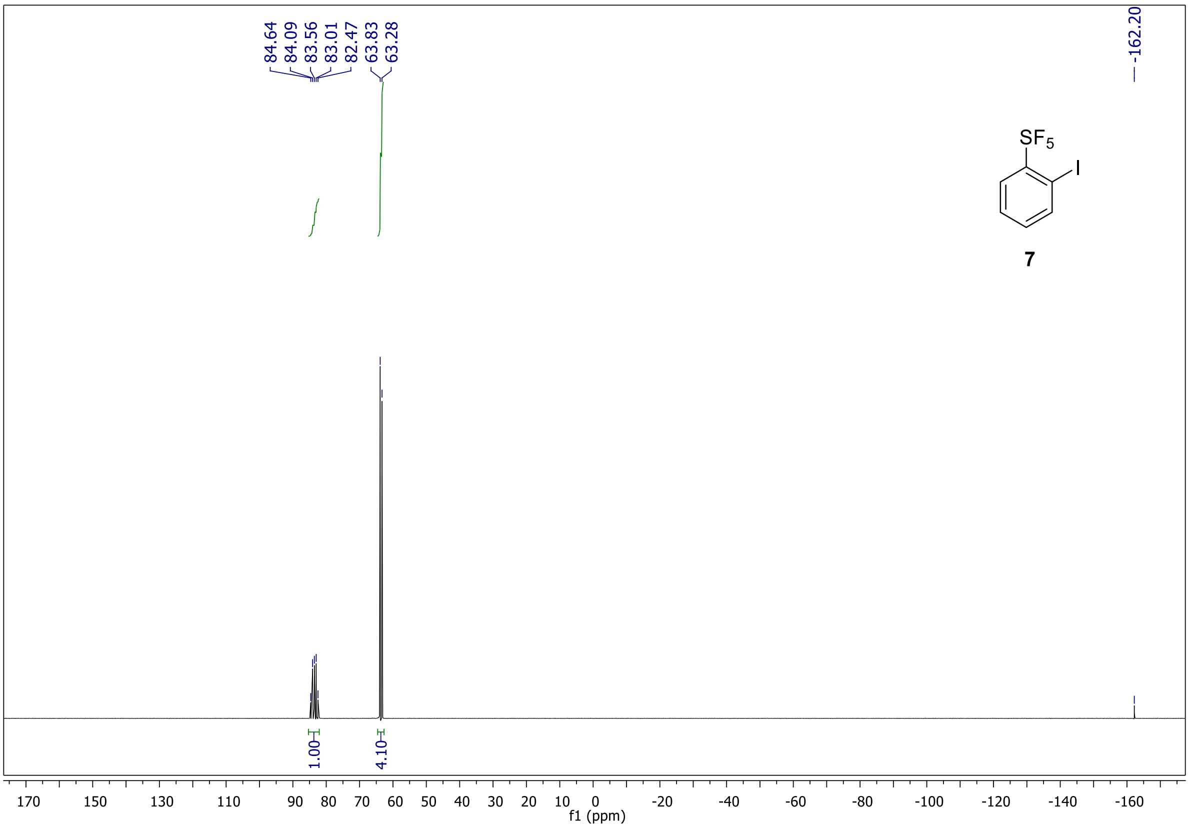
84.64
84.09
83.56
83.01
82.47
63.83
63.28
-162.20
SF5
I
7
1.00
4.10
f1 (ppm)

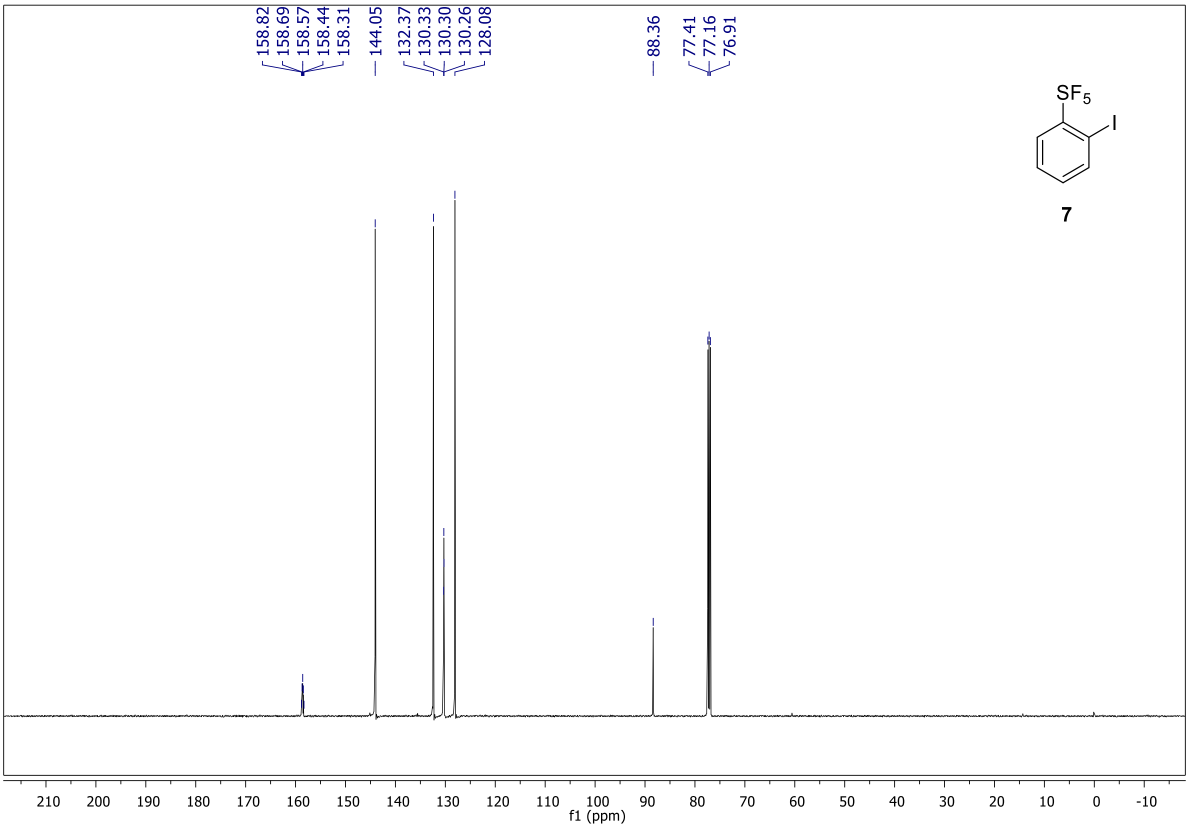
158.82
158.69
158.57
158.44
158.31
144.05
132.37
130.33
130.30
130.26
128.08
88.36
77.41
77.16
76.91
SF5
I
7
210 200 190 180 170 160 150 140 130 120 110 100 90 80 70 60 50 40 30 20 10 0 -10
f1 (ppm)

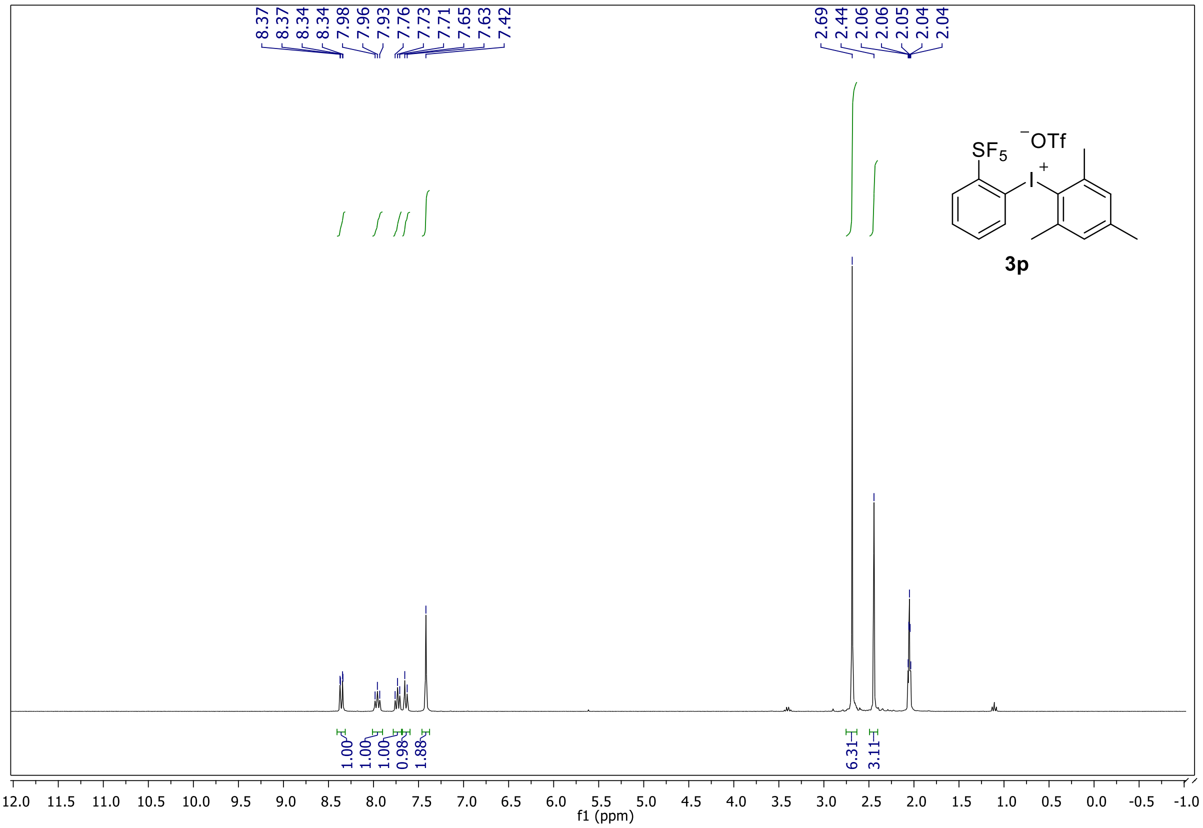
8.37
8.37
8.34
8.34
7.98
7.96
7.93
7.76
7.73
7.71
7.65
7.63
7.42
2.69
2.44
2.06
2.06
2.05
2.04
2.04
SF5
−OTf
I+
3p
1.00
1.00
1.00
0.98
1.88
6.31
3.11
12.0 11.5 11.0 10.5 10.0 9.5 9.0 8.5 8.0 7.5 7.0 6.5 6.0 5.5 5.0 4.5 4.0 3.5 3.0 2.5 2.0 1.5 1.0 0.5 0.0 -0.5 -1.0
f1 (ppm)

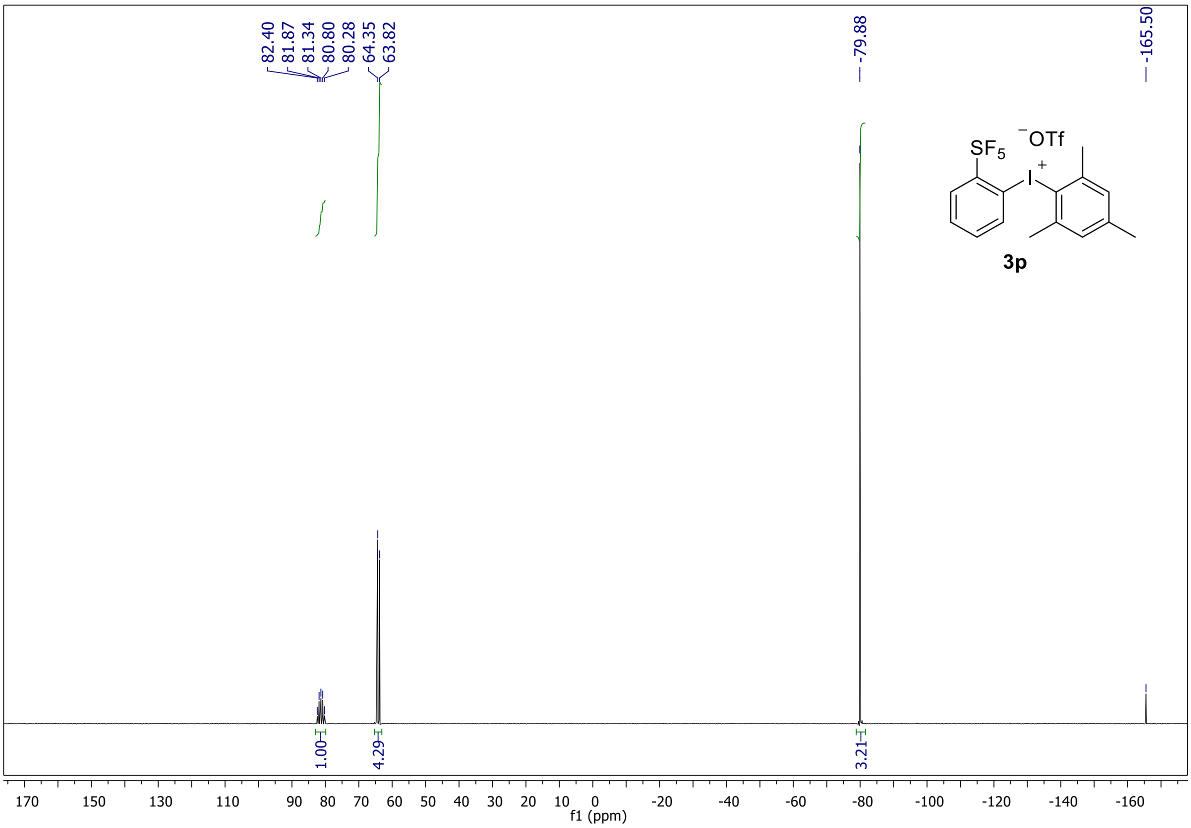
82.40
81.87
81.34
80.80
80.28
64.35
63.82
-79.88
-165.50
1.00
4.29
3.21
SF5
OTf
I+
3p
f1 (ppm)

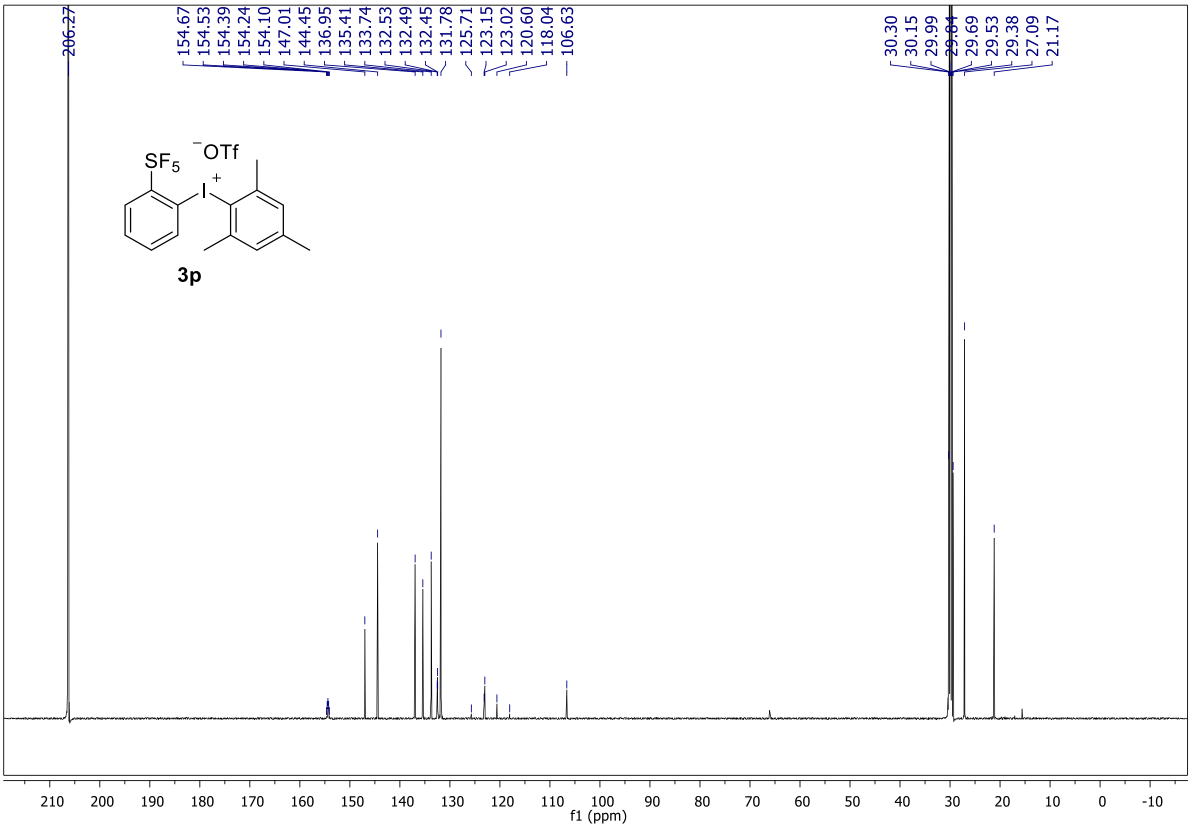
206.27
154.67
154.53
154.39
154.24
154.10
147.01
144.45
136.95
135.41
133.74
132.53
132.49
132.45
131.78
125.71
123.15
123.02
120.60
118.04
106.63
30.30
30.15
29.99
29.84
29.69
29.53
29.38
27.09
21.17
SF5
OTf
I+
3p
f1 (ppm)

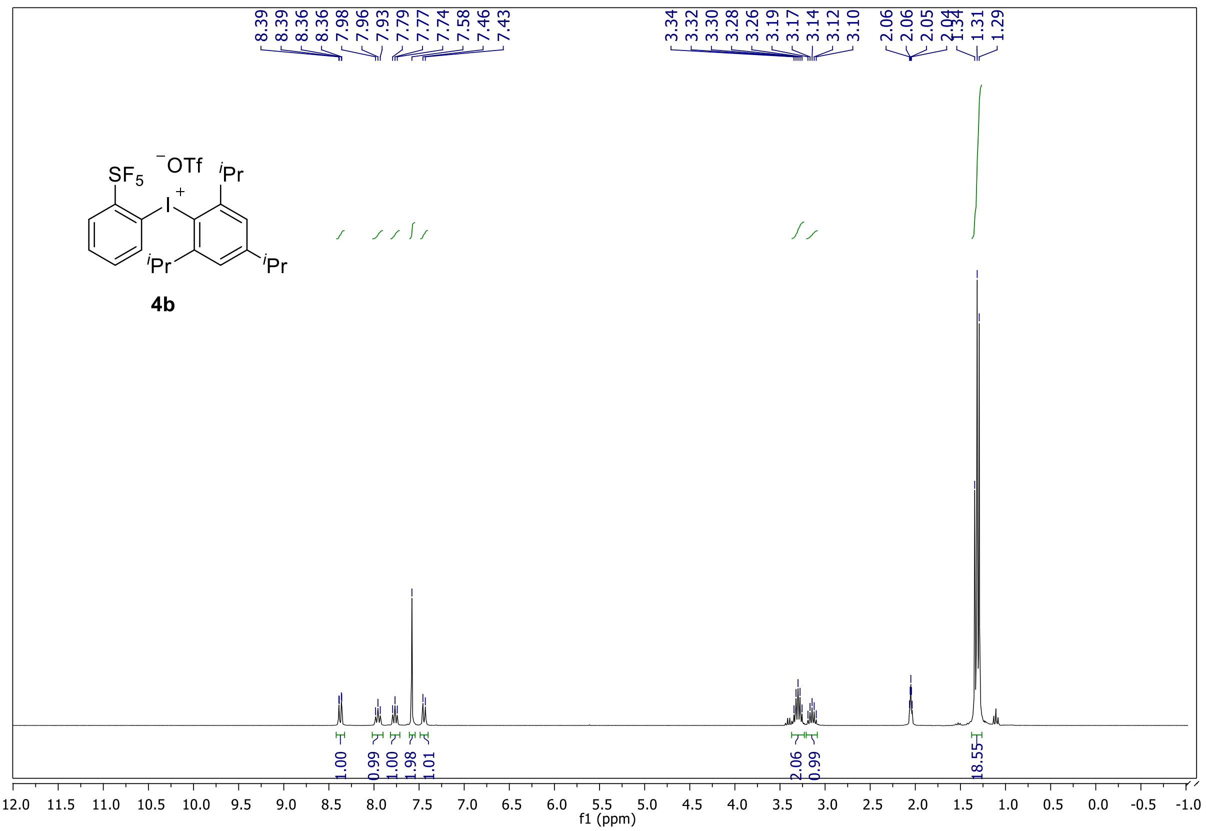
8.39
8.39
8.36
8.36
7.98
7.96
7.93
7.79
7.77
7.74
7.58
7.46
7.43
3.34
3.32
3.30
3.28
3.26
3.19
3.17
3.14
3.12
3.10
2.06
2.06
2.05
2.04
1.34
1.31
1.29
SF5
−OTf
+
I
iPr
iPr
iPr
4b
1.00
0.99
1.00
1.98
1.01
2.06
0.99
18.55
12.0 11.5 11.0 10.5 10.0 9.5 9.0 8.5 8.0 7.5 7.0 6.5 6.0 5.5 5.0 4.5 4.0 3.5 3.0 2.5 2.0 1.5 1.0 0.5 0.0 -0.5 -1.0
f1 (ppm)

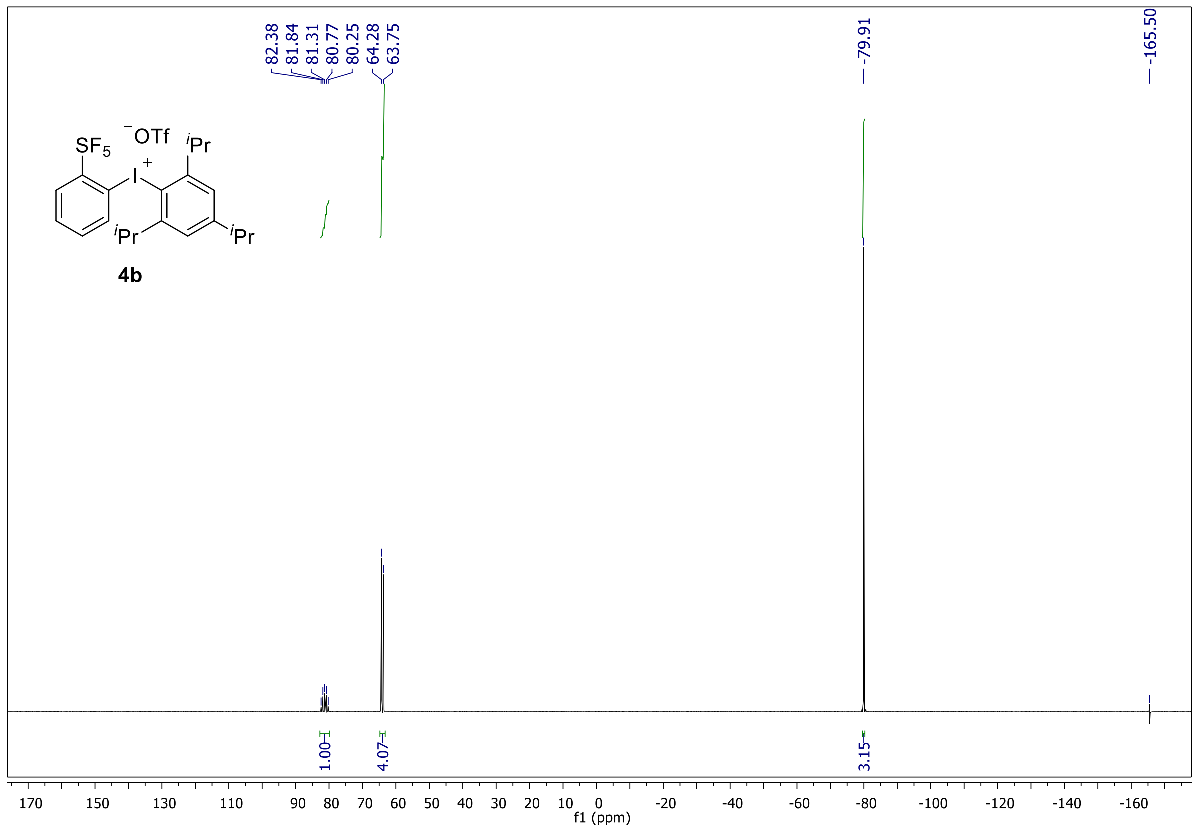
82.38
81.84
81.31
80.77
80.25
64.28
63.75
-79.91
-165.50
SF5
−OTf
+
I
iPr
iPr
iPr
4b
1.00
4.07
3.15
170 150 130 110 90 80 70 60 50 40 30 20 10 0 -20 -40 -60 -80 -100 -120 -140 -160
f1 (ppm)

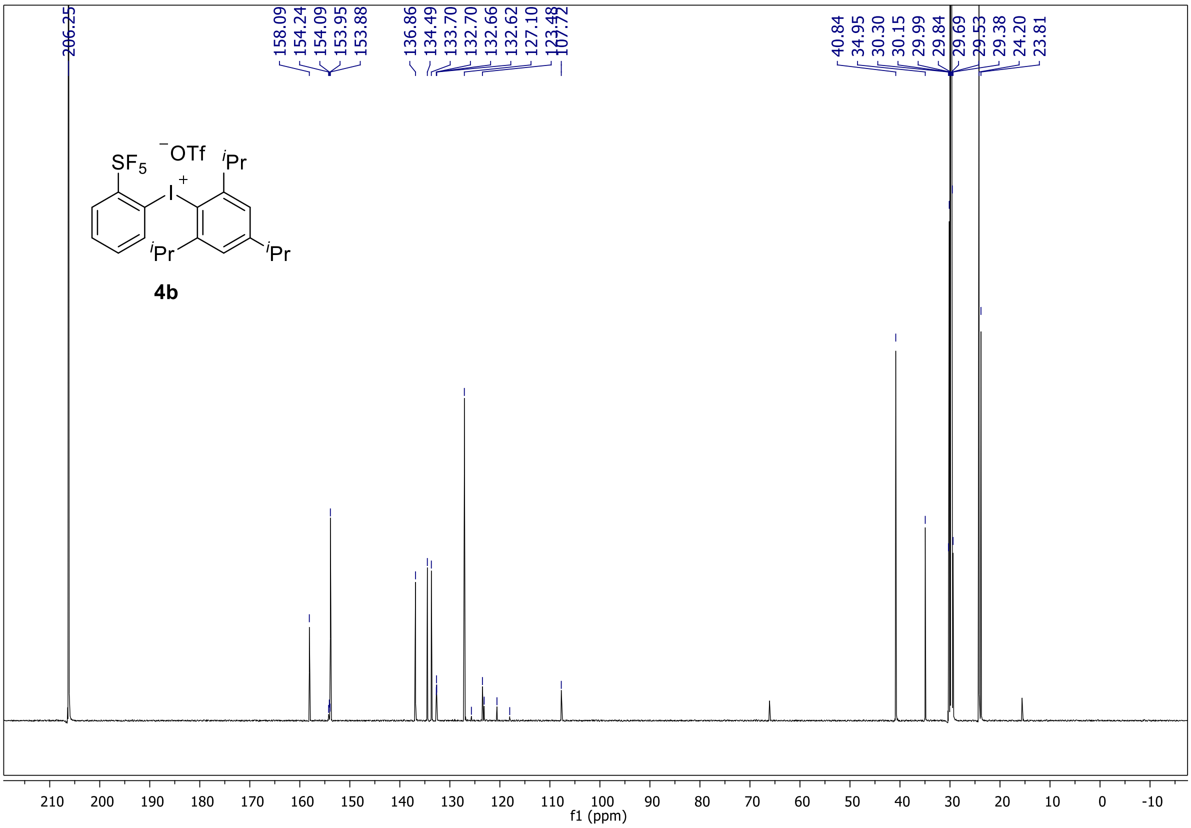
206.25
158.09
154.24
154.09
153.95
153.88
136.86
134.49
133.70
132.70
132.66
132.62
127.10
123.48
107.72
40.84
34.95
30.30
30.15
29.99
29.84
29.69
29.53
29.38
24.20
23.81
SF5
−OTf
iPr
I+
iPr
iPr
4b
f1 (ppm)

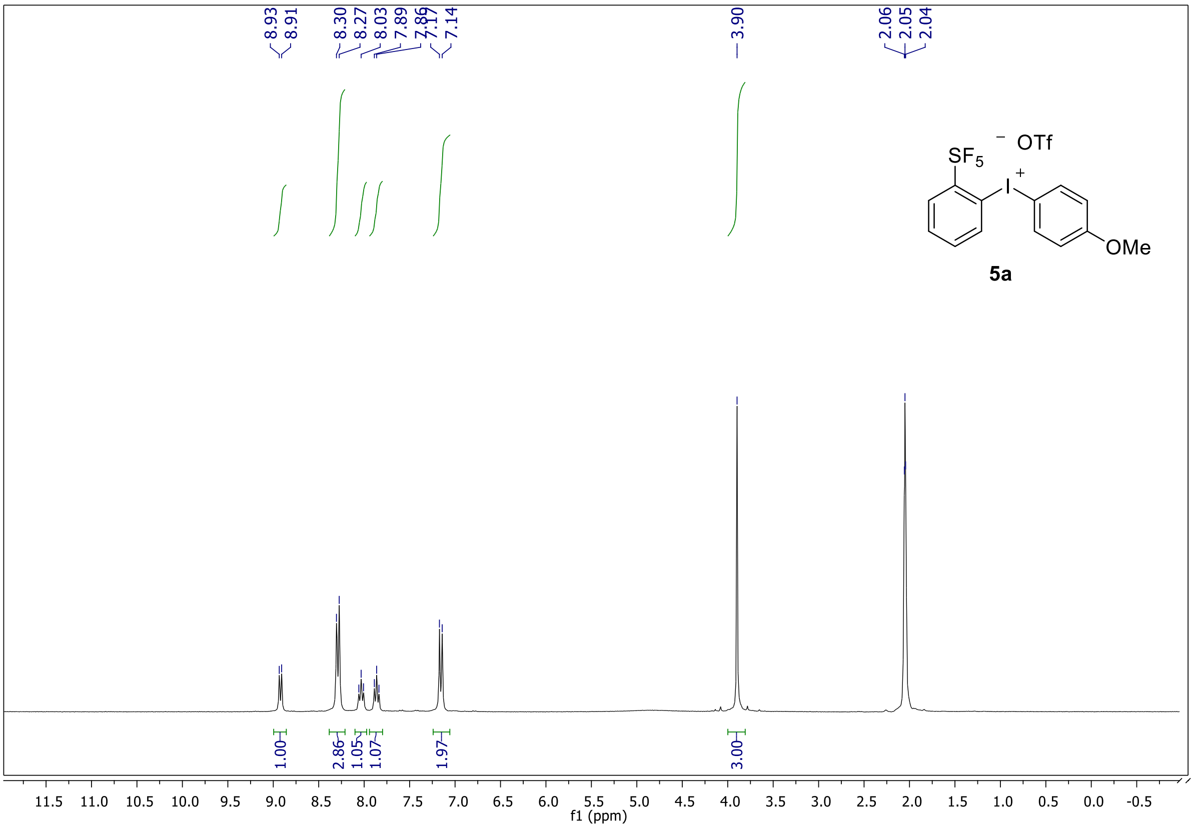
8.93
8.91
8.30
8.27
8.03
7.89
7.86
7.17
7.14
3.90
2.06
2.05
2.04
SF5
− OTf
I+
OMe
5a
1.00
2.86
1.05
1.07
1.97
3.00
f1 (ppm)

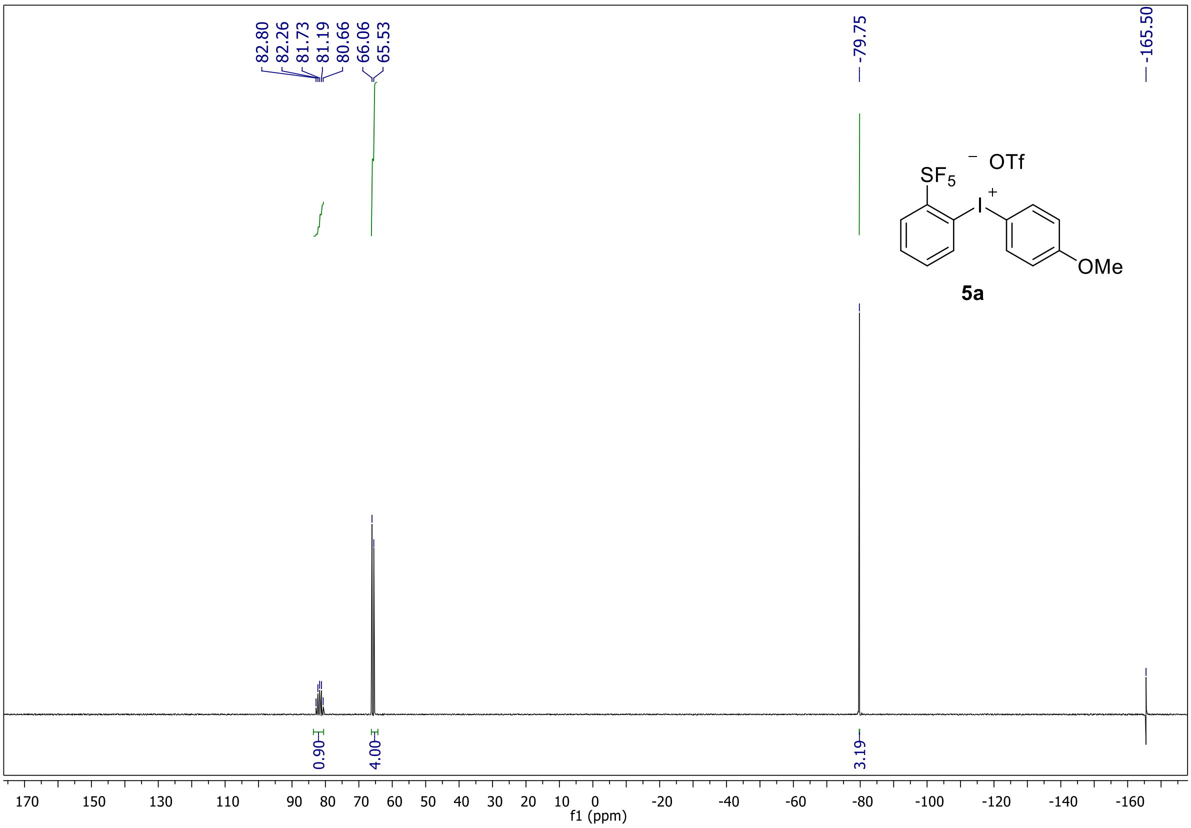
82.80
82.26
81.73
81.19
80.66
66.06
65.53
-79.75
-165.50
SF5
OTf
OMe
5a
0.90
4.00
3.19
170 150 130 110 90 80 70 60 50 40 30 20 10 0 -20 -40 -60 -80 -100 -120 -140 -160
f1 (ppm)

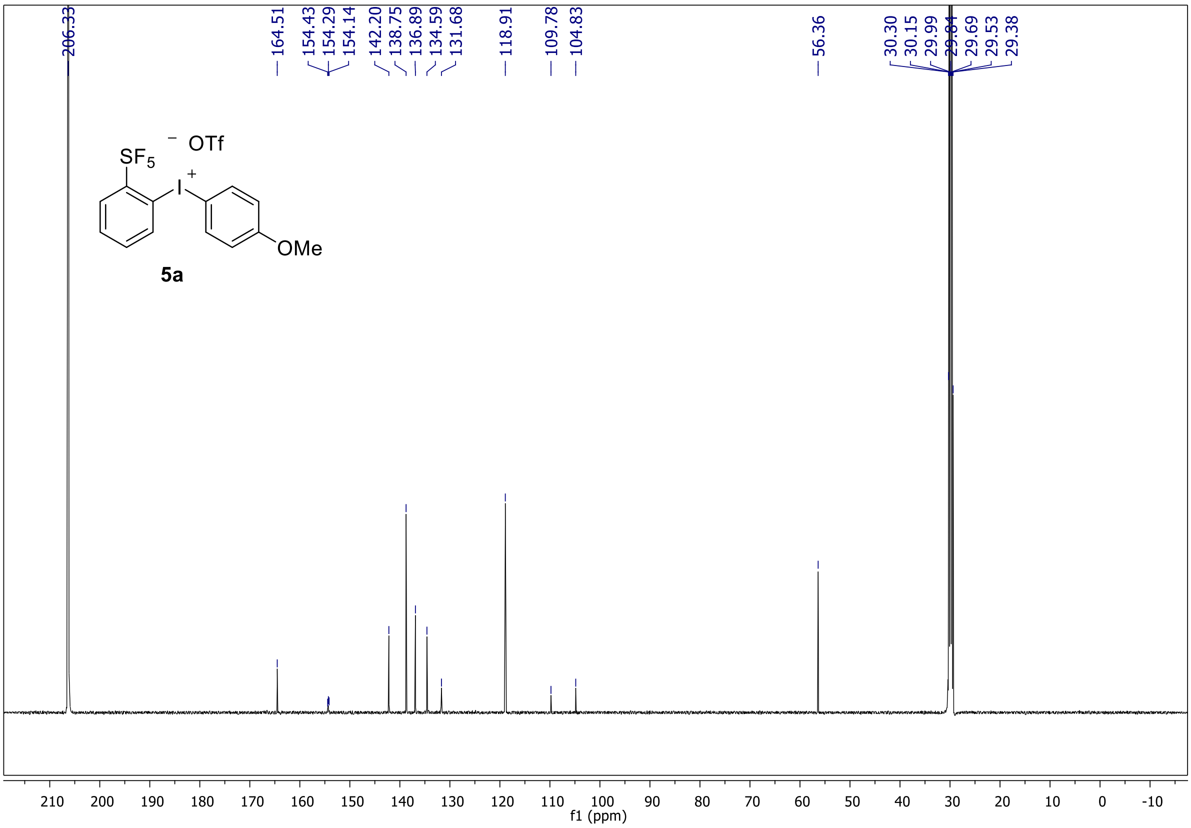
206.33
164.51
154.43
154.29
154.14
142.20
138.75
136.89
134.59
131.68
118.91
109.78
104.83
56.36
30.30
30.15
29.99
29.84
29.69
29.53
29.38
SF5
OTf
OMe
5a
210 200 190 180 170 160 150 140 130 120 110 100 90 80 70 60 50 40 30 20 10 0 -10
f1 (ppm)

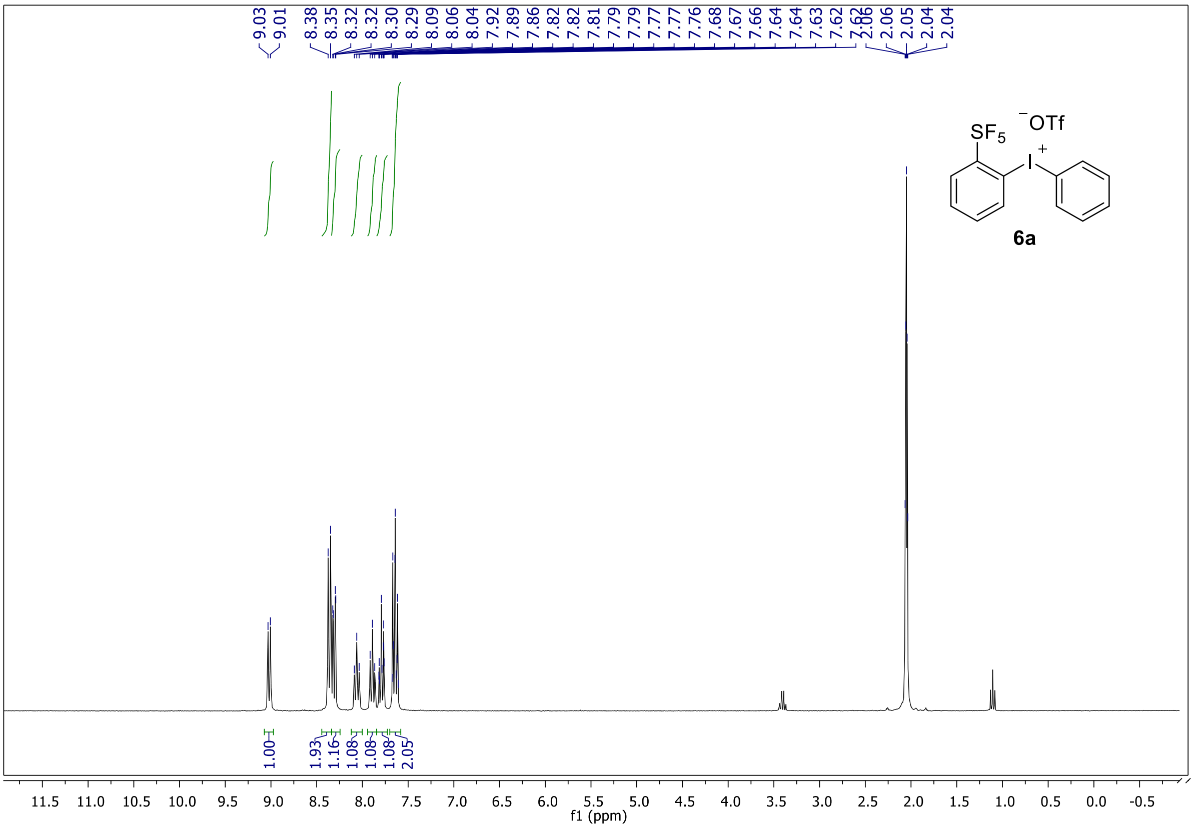
9.03
9.01
8.38
8.35
8.32
8.32
8.30
8.29
8.09
8.06
8.04
7.92
7.89
7.86
7.82
7.82
7.81
7.79
7.79
7.77
7.77
7.76
7.68
7.67
7.66
7.64
7.64
7.63
7.62
7.62
2.06
2.05
2.04
2.04
SF5
−OTf
I+
6a
1.00
1.93
1.16
1.08
1.08
1.08
2.05
11.5 11.0 10.5 10.0 9.5 9.0 8.5 8.0 7.5 7.0 6.5 6.0 5.5 5.0 4.5 4.0 3.5 3.0 2.5 2.0 1.5 1.0 0.5 0.0 -0.5
f1 (ppm)

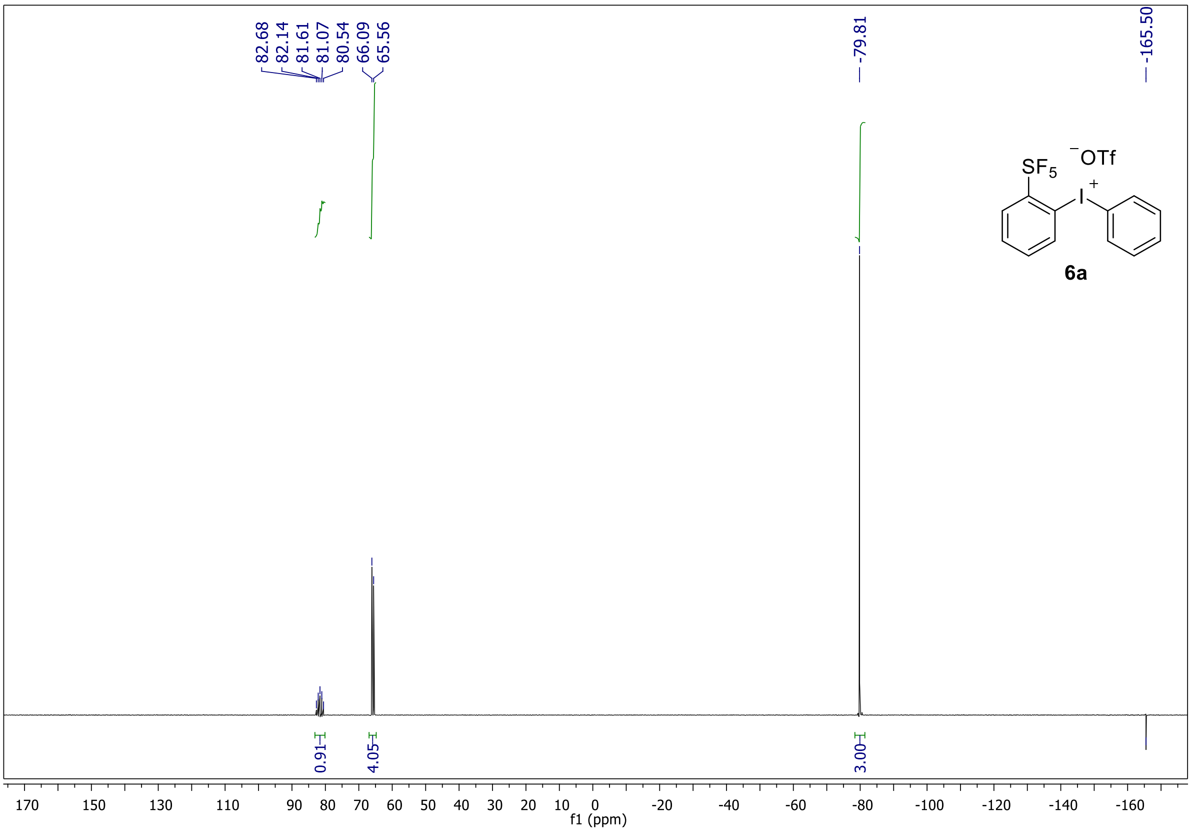
82.68
82.14
81.61
81.07
80.54
66.09
65.56
-79.81
-165.50
SF5
−OTf
I+
6a
0.91
4.05
3.00
170 150 130 110 90 80 70 60 50 40 30 20 10 0 -20 -40 -60 -80 -100 -120 -140 -160
f1 (ppm)

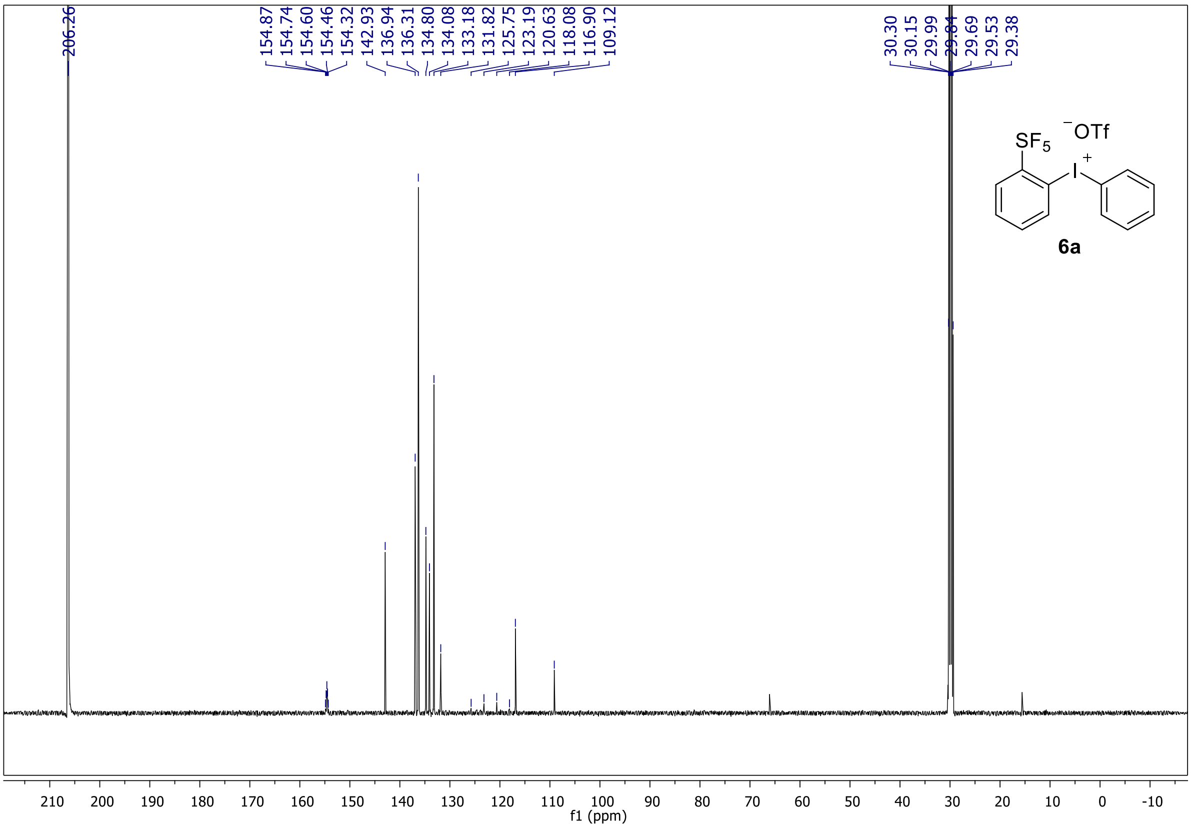
206.26
154.87
154.74
154.60
154.46
154.32
142.93
136.94
136.31
134.80
134.08
133.18
131.82
125.75
123.19
120.63
118.08
116.90
109.12
30.30
30.15
29.99
29.84
29.69
29.53
29.38
SF5
⁻OTf
I+
6a
210 200 190 180 170 160 150 140 130 120 110 100 90 80 70 60 50 40 30 20 10 0 -10
f1 (ppm)
